# Annual Research Review: The impact of Covid‐19 on psychopathology in children and young people worldwide: systematic review of studies with pre‐ and within‐pandemic data

**DOI:** 10.1111/jcpp.13716

**Published:** 2022-11-24

**Authors:** Tamsin Newlove‐Delgado, Abigail Emma Russell, Frances Mathews, Lauren Cross, Eleanor Bryant, Rebecca Gudka, Obioha C. Ukoumunne, Tamsin J. Ford

**Affiliations:** ^1^ University of Exeter Medical School University of Exeter Exeter UK; ^2^ Department of Psychiatry University of Cambridge Cambridge UK; ^3^ NIHR Applied Research Collaboration South West Peninsula (PenARC) Exeter UK

**Keywords:** Covid‐19, pandemic, children, young people, mental health

## Abstract

**Background:**

The high volume and pace of research has posed challenges to researchers, policymakers and practitioners wanting to understand the overall impact of the pandemic on children and young people's mental health. We aimed to search for and review the evidence from epidemiological studies to answer the question: how has mental health changed in the general population of children and young people?

**Methods:**

Four databases (Medline, CINAHL, EMBASE and PsychINFO) were searched in October 2021, with searches updated in February 2022. We aimed to identify studies of children or adolescents with a mean age of 18 years or younger at baseline, that reported change on a validated mental health measure from prepandemic to during the pandemic. Abstracts and full texts were double‐screened against inclusion criteria and quality assessed using a risk of bias tool. Studies were narratively synthesised, and meta‐analyses were performed where studies were sufficiently similar.

**Results:**

6917 records were identified, and 51 studies included in the review. Only four studies had a rating of high quality. Studies were highly diverse in terms of design, setting, timing in relation to the pandemic, population, length of follow‐up and choice of measure. Methodological heterogeneity limited the potential to conduct meta‐analyses across studies. Whilst the evidence suggested a slight deterioration on some measures, overall, the findings were mixed, with no clear pattern emerging.

**Conclusions:**

Our findings highlight the need for a more harmonised approach to research in this field. Despite the sometimes‐inconsistent results of our included studies, the evidence supports existing concerns about the impact of Covid‐19 on children's mental health and on services for this group, given that even small changes can have a significant impact on provision at population level. Children and young people must be prioritised in pandemic recovery, and explicitly considered in planning for any future pandemic response.

## Introduction

The Covid‐19 pandemic has affected the daily lives and experiences of children across the globe. Over the course of the pandemic, UNICEF estimates that 99% of the world's 2.36 billion children lived in a country that imposed at least some movement restrictions, with more than half experiencing some form of lockdown (UNICEF, 2021). The most obvious example of these changes to daily lives is the effect on education, with the response to Covid‐19 leading to an estimated 1.6 billion children being out of school for an extended period (Dreesen et al., 2020). However, an extensive range of wider impacts, both positive and negative, have been recognised on relationships, family and community life, as well as on economic prosperity. For the individual child, these will interact with existing circumstances, vulnerabilities and protective factors, and have been described as vicious and virtuous cycles created by Covid‐19 (Calvano et al., 2021; Creswell et al., 2021; Gadermann et al., 2021).

Initially, concerns around the impact on children centred around the experience with previous pandemics, and on the potential for post‐traumatic symptoms in children following short and strict lockdowns in some parts of the world. As it became apparent that the acute response and the restrictions to daily lives would last more than a few weeks, the potential for more extensive impacts became apparent, leading to a proliferation of studies using convenience samples and sometimes newly developed ‘pandemic impact’ measures, attempting to provide rapid answers to questions about the impact on mental health. Such research was able to provide quick snapshots of the mental health or wellbeing of those who responded but is significantly limited in terms of capturing impact on the more underserved or digitally unengaged populations, as well as individual trajectories over time. Early during the pandemic, Pierce et al. (2020) raised concerns over drawing conclusions from convenience samples, arguing ‘the investigators should use rigorous methods that sample from the whole population to reduce erroneous conclusions and potentially damaging actions’. Similarly, during 2021, the DEPRESSD living systematic review screened over 45,000 papers on the impact of Covid‐19 on mental health, noting that the ‘rapid pace, high volume, and limited quality of mental health evidence being generated during COVID‐19 poses a barrier to effective decision‐making’ (Sun et al., 2021).

High‐quality evidence on changes in child mental health remains relevant in informing shorter‐term prevention and recovery efforts, such as support for families and in schools, and addressing waiting lists for services. It is also crucial in setting policy priorities to address and mitigate medium‐ and longer‐term societal and economic impacts, such as on child development, education and employment opportunities, and community and social cohesion, which are likely to compound existing inequalities (Kyeremateng, Oguda, & Asemota, 2022). For example, the Mental Health of Children and Young People in England surveys reported that children with probable mental health disorders were more likely to live in households that had fallen behind with bills, rent or mortgage during the pandemic, or to live with food insecurity (Newlove‐Delgado et al., 2021).

Evidence from research on harms and benefits is also relevant in informing future response to pandemics in terms of restrictions and public health protections, and where support and mitigations may need to be targeted. For example, the policies on school closures and subsequently on public health measures in schools have been extensively debated in terms of the mental health, educational and developmental impacts, but also considering impacts on controlling transmission, inclusivity for children and families who might be clinically vulnerable and, more recently, the risks of Long Covid in children (Stephenson et al., 2022; Viner et al., 2021). For governments, decisions involve balancing complex considerations on risks and benefits and therefore should be informed by the best possible evidence.

Our brief was to search for and review the evidence from epidemiological studies which include population samples, prepandemic data and validated mental health measures. Our research questions is: how has mental health changed in the general population of children and young people, comparing measures of mental health prepandemic with measures during the Covid‐19 pandemic?

Whilst this does not represent the first systematic review on this topic, the focus on population samples and validated mental health measures, including externalising symptoms, complements and extends previous reviews, such as that by Bussières et al. (2021). Through this review, we have aimed to assess breadth and quality of existing research, provide an authoritative overview of the impacts on mental health from high‐quality research, and identify where future efforts should be concentrated.

## Methods

This systematic review aimed to review studies of children or adolescents with a mean age of 18 years or younger at baseline, that report change in mental health from prepandemic to during the pandemic using the same measure.

### Eligibility criteria

Eligible study designs were longitudinal cohort studies (where a cohort was assembled prepandemic, with at least one follow‐up wave during the pandemic) or cross‐sectional studies with follow‐ups (where participants in a cross‐sectional survey prepandemic were followed up with one or more survey waves during the pandemic). We also included studies which compared samples from different cohort or cross‐sectional surveys from prepandemic to during‐pandemic, if they demonstrated that the populations were comparable. Studies need to have a baseline (prepandemic) validated mental health measure collected within 5 years prior to the onset of the pandemic (December 2014–December 2019), and a follow‐up measure between January 2020 and February 2022, when update searches were completed.

Studies were included if they reported change on a validated measure of mental health aligning with Diagnostic and Statistical Manual of Mental Disorders Fifth Edition (DSM‐5; Diagnostic and statistical manual of mental disorders: DSM*‐*5™, 2013) or International Classification of Diseases 11th Revision (ICD‐11; International statistical classification of diseases and related health problems, 2019) criteria for mental health or neurodevelopmental disorders. Examples include the Strengths and Difficulties Questionnaire (SDQ; Goodman, 1997), Child Behaviour Checklist (CBCL; Pedersen et al., 2021) and the Center for Epidemiologic Studies Depression Scale (CES‐D; Radloff, 1977). These measures could be reported by the child or young person, parents or guardians, teachers, or clinicians. We also included routine data using a population sample where appropriate, for example, reporting of suicide via the National Child Mortality Database in England (Odd, Williams, Appleby, Gunnell, & Luyt, 2021). Given the focus on mental health at a population level, we excluded routine data collected by services, such as hospital admissions, prescriptions issued and recording of diagnoses by clinicians. Measures of wellbeing and constructs related to mental health were excluded (e.g. stress, loneliness and anger). Studies altering validated measures or developing bespoke measures were excluded, and those only measuring change in mental health during the pandemic were also excluded. Mental health measures could be reported on a continuous scale such as change in mean SDQ total difficulties or number of symptoms, or could be reported categorically, such as the change in proportion of those meeting cut‐offs on a scale.

All primary research and analyses of existing data were eligible for inclusion if published in a peer‐reviewed journal, but editorials, commentaries, reviews and other nonprimary research were excluded. Where conference abstracts contained sufficient data to meet inclusion criteria, they were eligible to be included. Searches were conducted to identify if further information had been published subsequent to the conference.

Eligible study samples were from the general population (from settings representative of the general population or using probability sampling) or from education settings, such as schools and colleges. Samples from trials were also eligible if these recruited from the general population or education settings, such as prevention trials. Representativeness of the sample and sampling approach (e.g. probability sample and convenience sample) was assessed as part of our quality and risk of bias assessment. Studies could be in any country but were only included if published in English. Studies of University or further education students which focussed on those on specific courses were excluded as we considered their experience of the pandemic may vary from the general population given their studies (for instance, medical or nursing students). Similarly, samples of young people presenting to clinical services were also excluded due to potential bias in accessing and seeking mental health support.

### Search strategy

Four databases (Medline, CINAHL, EMBASE and PsycINFO) were searched in October 2021, with searches updated at the end of February 2022. Table S1 shows the search strategy in Medline. Search terms included terms relating to Covid‐19, terms relating to children and adolescents, and terms relating to mental health and mental illness. Date filters were used to identify studies published since 2019, given that no studies prior to this would have during‐pandemic measures and would thus be ineligible.

### Selection process

Following searches of all databases, duplicate records were identified and removed. In the first stage of screening, titles and abstracts were screened by two independent authors. Following this, full texts were retrieved and then also screened by two independent authors to determine the final list of studies meeting inclusion criteria. Cadima (https://www.cadima.info/) was used to manage the screening and study selection process. Discrepancies in ratings were discussed and resolved through individual discussion and weekly team meetings, with TND and AR making final decisions on inclusion. We registered our review protocol on PROSPERO (CRD42021293296) before beginning data extraction.

### Data extraction

A data extraction form was developed that captured:

1. Study characteristics (reference details, country, study design and name of cohort/sample if applicable, the date of first restrictions relating to Covid‐19 as reported by the authors). Where authors did not report this information, we used the Financial Times *Tracking Covid‐19* interactive map (Bernard, 2022), which reports restrictions worldwide by country and date from 23 January 2020 using the Oxford Covid‐19 government response stringency index (Hale et al., 2021; range 0–100). Data on the stringency of lockdown at the time that follow‐up data collection began were extracted for each study.
2. Study population characteristics at baseline (prepandemic), including sampling, number approached, eligible, recruited and the overall response rate, the mean age and age range of the sample at baseline, details on participant sex and ethnicity, dates of baseline data collection and mode (e.g. face‐to‐face and online survey).
3. Details of follow‐up data collection, with any differences from baseline noted (e.g. face‐to‐face data collection switching to online survey). We also extracted the characteristics of the follow‐up sample if this was different to the baseline sample, such as from a different cohort or cross‐sectional study. The range in months from the earliest baseline data collection to latest follow‐up, and vice‐versa (latest baseline to earliest possible follow‐up) was also extracted for each study.
4. Detailed information on mental health measures, informants and any data and statistical information that captured or assessed change in mental health from baseline to follow‐up (pre‐ to during‐pandemic) for each measure and subscale (including but not limited to change in mean scores and change in proportion meeting a defined cut‐off), as well as summarising key findings. Where studies reported findings by subgroups we also extracted relevant data for each subgroup analysis (e.g. by participant sex or ethnicity). Data were extracted by one author for each study, with 20% of studies being checked by TND and AR. Extractions by the two most junior members of the team were additionally checked for accuracy by other team members.

### Appraisal of risk of bias and quality

A modified version of Joanna Briggs Institute critical appraisal tools was used to assess the quality of included studies in terms of their risk of bias, based on the cohort and cross‐sectional studies checklists (Joanna Briggs Institute, 2017). AR and TND conducted the critical appraisal, first appraising four included studies independently, then discussing and resolving discrepancies prior to each appraising half of the remaining studies. Each study was rated on the following: (a) Was the sampling and recruitment clearly described? (b) Was the sample representative of the general population of children and young people? (c) If a comparison of two different cohorts, were the characteristics of participants sufficiently similar? (d) Was loss to follow‐up described? (e) Were any strategies used to address incomplete follow‐up? (f) Was statistical analysis appropriate? These answers were used to assess whether a study was at low, medium, high, or unclear risk of bias in each of these domains. The individual study ratings are displayed in Figure S1, using the *robvis* risk of bias visualisation tool (McGuinness & Higgins, 2021). Based on these ratings and any other concerns there might be about quality, we provided an overall quality rating for each study. We assigned an overall quality rating of ‘high’ where all risk of bias items were judged as being low, and where there were no other concerns about the study methodology or reporting. Where there were unclear, medium or high risk of bias ratings on any items for a study this was assigned as ‘medium/high’, ‘medium’, ‘medium/low’ or ‘low’ quality, depending on the nature and extent of the potential biases, and on other factors identified. These overall individual study assessments are displayed in Table 1.

**Table 1:** Summary of study characteristics

First author	Design	Country and setting	Time between baseline and pandemic data collection	Restriction stringency at pandemic data collection^a^	Age at baseline (year)	*N* at follow‐up	Quality rating	Measures	Mental health outcome domain
					Mean (range)				
Ravens‐Sieberer et al. (2022)	Cross‐cohort comparison	Germany	40–41 months	Medium	12.5 (7–17)	2626	M	SDQ SCARED CES‐DC	Total difficulties Depression symptoms
De France et al. (2021)	Cohort	Canada: community	41–42 months	High	13.9	136	M	CDI MASC	Depression symptoms Anxiety symptoms
Gladstone et al. (2021)	Cross‐sectional with follow‐up	USA	4–7 months	High	14.5 (12–18)	228	M	PHQ‐9	Depression symptoms
Magson et al. (2021)	Cohort	Australia	6–17 months	High	13.4	248	L	SMFQ, Brief Spence Anxiety	Depression symptoms Anxiety symptoms
Mehus et al. (2021)	Cross‐sectional with follow‐up	USA	3–4 months	Very low to high	18.6 (18–20)	727	M	GAD‐7 PHQ‐9	Depression symptoms Anxiety symptoms
Chen et al. (2021)	Cohort	China	5–6 months	High to very high	10.32	543	L/M	DASS‐21	Depression symptoms Anxiety symptoms
Feinberg et al. (2021)	Cohort	USA: parenting trial	4–39 months	High	9.9 at f/u	208	M	SDQ	Externalising and internalising symptoms
Daniunaite et al. (2021)	Cohort	Lithuania: schools	17–19 months	Low to medium	12–16	331	L/M	SDQ	Prosocial skills Peer relationship problems Emotional symptoms Conduct problems Hyperactivity/ inattention
Hafstad et al. (2021)	Cohort	Norway	16–17 months	Medium	12–16	3572	M/H	HSCL‐10	Combined/global difficulties
Wright et al. (2021)	Cohort	UK	3–7 months	High	10–12	202	H	SMFQ Brief Spence Anxiety CBCL Child Trauma Scale	Depression symptoms Anxiety symptoms Trauma
Browne et al. (2021)	Cohort	Canada	3–4 months	Low to high	3–12	214	M	SDQ	Total difficulties
Jolliff et al. (2021)	Comparison of cross‐sectional survey samples	USA: community	1–6 months	Low to high	14.7	134	L/M	GAD‐7 PHQ‐8	Depression symptoms Anxiety symptoms
Hanno et al. (2021)	Cohort	USA: community	18 months	High	2.5–5	567	M/H	BASC BRIEF‐P	Internalising symptoms Externalising symptoms
Hu and Qian (2021)	Cohort, probability sample	UK: community	3–18 months	High	10–16	886	M/H	SDQ	Prosocial skills Peer relationship problems Emotional symptoms Conduct problems Hyperactivity/ inattention
Burdzovic Andreas and Brunborg (2021)	Cohort	Norway: schools	12–24 months	Low to medium	16	915	M	PHQ‐9	Depression symptoms
Liao et al. (2021)	Cohort: cluster randomised	China: schools	6–7 months	High	11–16	2496	M/H	CES‐DC	Depression symptoms
Walters et al. (2021)	Cross sectional with follow‐up	USA: schools	12 months	High	12.5	170	L	CES‐D Weinberger Adjustment Inventory Impulse Control scale	Depression symptoms Impulsivity
Hamza et al. (2021)	Cross‐sectional with follow‐up	Canada: schools/colleges	12 months	High	18.5	733	L	PSS‐10 PNAS Inventory of Statements about Self‐Injury, CES‐D MSI‐BPD AUDIT PTSD Checklist GAD‐7	Self‐harm BPD symptoms Depression symptoms Anxiety symptoms
Myhr et al. (2021)	Comparison of cross‐sectional surveys	Norway: schools	2–3 months	Medium to high	13–16	1957	L	Depression Mood Inventory	Depression symptoms
Ezpeleta et al. (2020)	Cohort	Spain: community	Unclear ~12 months	Medium	12	226	L/M	SDQ	Total difficulties Prosocial skills Peer relationship problems Emotional symptoms Conduct problems Hyperactivity/ inattention
Westrupp et al. (2021)	Comparison of cross‐sectional surveys	Australia	4.5–5 years	High	9 (0–18)	2365	M/H	SMFQ	Depression symptoms
Li et al. (2021)	Cross‐sectional with follow‐up	China: schools	6–7 months	High	14–19	831	M	Zung Self‐Rating Anxiety Scale. BDI	Depression symptoms Anxiety symptoms
Lane et al. (2022)	Comparison of cross‐sectional surveys	Canada: schools	12 months	High	12.6	1380	L	SCARDs‐R CDI	Depression symptoms Generalised anxiety Social anxiety Separation anxiety Panic disorder
Zhang et al. (2020)	Cohort	China: schools	6 months	Very high	12.6 (9–16)	1241	M	MFQ MacArthur HBQ, Youth Risk Behaviour Surveillance System	Depression symptoms Self‐harm and suicidal ideation
Khoury et al. (2021)	Cohort	Canada: mother‐child dyad study	2–4 years	High	5.2	68	L/M	BPM	Externalising symptoms Internalising symptoms
Hussong et al. (2021)	Cohort	USA: university staff/ community	3–4 years	High	6–9	88	L	Paediatric Symptom Checklist	Global difficulties
Bélanger et al. (2021)	Cohort	Canada: schools	12–24 months	High	11–17	1880	M/H	CES‐D GAD‐7	Depression symptoms Anxiety symptoms
Bignardi et al. (2020)	Cohort	England: schools	7–24 months	High	6–9	168	M	SDQ emotional problems subscale RCADS	Internalising symptoms Depression symptoms Anxiety symptoms
Halldorsdottir et al. (2021)	Comparison of cross‐sectional surveys	Iceland	2 years	Medium	14–15	523	L	SCL‐90	Depression symptoms
van der Laan et al. (2021)	Cohort	Netherlands: birth cohort	46–405 days (mean 10 months)	High	14.8 (12–16)	158	M/H	RCADS	Anxiety symptoms
Koenig et al. (2021)	Comparison of matched‐pair samples within the same cohort	Germany: PROHEAD prevention trial	5 days to 2 years	Medium	14.9 (12–20)	324	M	SDQ PHQ‐A Eating Disorder Examination ‐ Questionnaire (EDE‐Q)	Total difficulties Prosocial skills Peer relationship problems Emotional symptoms Conduct problems Hyperactivity/ Inattention Eating disorders Depression symptoms
Thorisdottir et al. (2021)	Comparison of repeated cross‐sectional surveys	Iceland: Youth in Iceland school surveys	4 years	Low to medium	13–18	14,475	M/H	SCL‐90 (depression subscale)	Depression symptoms
Luijten et al. (2021)	Comparison of cross sectional surveys, probability samples	Netherlands	22–30 months	High	13.4	844	H	PROMIS‐CAT V2 (depression and anxiety domains)	Depression symptoms Anxiety symptoms
Hollenstein et al. (2021)	Cohort	Canada	3–9 months	High	12.5 (12–13)	292	M	CDI BDI	Depression symptoms
Zhu et al. (2021)	Cross sectional with follow‐up	China: schools	9 months	High to very high	13.0 (10–17)	1,393	M	GAD‐7 PHQ‐8	Depression symptoms Anxiety symptoms
Metherell et al. (2021)	Cohort	UK: Understanding Society	2–3.5 years	High to very high	10‐15	836	M	SDQ	Total difficulties
Mlawer et al. (2022)	Cohort	US: schools	3–11 months	High	16	96	L	CDI MASC	Depression symptoms Anxiety symptoms
Odd et al. (2021)	Cross‐sectional comparison using National Child Mortality Database (NCMD) ‐ routine data collection	England: whole population	1 year	High	0–18	N/A	H	Routine data on suicide	Suicide
Shoshani and Kor (2022)	Cross sectional with follow‐up	Israel: schools	9 months	Very high to high	11.1–17	1,537	L	Brief Symptom Inventory 18	Depression symptoms Anxiety symptoms Global severity
Valdez‐Santiago et al. (2022)	Comparison of cross‐sectional surveys	Mexico	1 year	High	10–19	4,812	M/H	Child reported Last‐year suicide attempts	Suicide attempts
von Soest et al. (2022)	Comparison of repeated cross‐sectional surveys	Norway: national surveys	2 years	Medium to high	15.5	86,597	H	Depressive Mood Inventory	Depression symptoms
Wang et al. (2021)	Cohort	China: schools	1 year	Medium to high	14.4	1,790	M/H	GAD‐7 CES‐D	Anxiety symptoms Depression symptoms
Widnall et al. (2021) (Abstract)	Cross sectional with follow‐up	UK: schools	8 months	High	13–17	603	U	HADS	Anxiety symptoms Depression symptoms
Adachi et al. (2022)	Cohort	Japan: schools	10 months	Low	9–12	4,875	L/M	PHQ‐A	Depression symptoms
Ertanir et al. (2021)	Cohort	Switzerland: schools	12 months	Medium	12.6	319	L	HSCL‐25	Depression symptoms
Frigerio et al. (2022)	Cohort	Italy: birth cohort	1 year	Very high to high	3.5	59	L	CBCL (Anxiety/depression and attention problems subscales)	Internalising symptoms Attention problems
Gadassi Polack et al. (2021)	Cohort	USA: community/university	6–17 months	High	9–15	112	L/M	CDI	Depression symptoms
Gimenez‐Dasi et al. (2021)	Cross sectional with follow‐up	Spain: schools	2–5 months	High	6–11	66	L	SENA anxiety scale	Anxiety symptoms
He et al. (2022)	Cohort	China	12 months	Medium to high	12.5	1687	M	CES‐DC	Depression symptoms
Howard et al. (2022)	Cohort	Canada: university	7 months	High	18.4	411	M	CED‐D 10 GAD 7	Anxiety symptoms Depression symptoms
Liu et al. (2022)	Cohort	USA	1 week to 5 years	High	16.1 (f/u)	175	M	CDI 12	Depression symptoms

^a^Restinction stringency based on the Oxford Covid‐19 government response stringency index.

### Synthesis methods

We synthesised and reported our findings by domain of mental health. After creating a detailed table of study characteristics (presented in summary form in Table 1), we assessed whether any studies were sufficiently homogenous to meta‐analyse in terms of using the same measure, which was reported in such a way that findings could be meaningfully synthesised.

Given the wide variation in included studies, we conducted meta‐analyses where there were: more than one study using the same measure, by the same informant, and using the same broad design (with separate meta‐analyses for studies including different participants at baseline and follow‐up, and those following the same participants over time), reporting sample size, mean and standard deviation of the measure at baseline and follow‐up, or the mean difference with standard error or confidence intervals. Mean changes and standard errors were calculated from the data extracted from the papers using the *ttesti* command in Stata v17 (StataCorp, 2021), and these were then entered into random effects meta‐analysis as standardised mean differences. The *I* squared (*I* ^2^) statistic was reported to quantify statistical heterogeneity in the study results. We do not account for the correlation between the measures on the same participants over time for cohort studies, which means the confidence intervals for our pooled effect estimates are conservatively wide.

Narrative syntheses were used to complement and extend the findings from meta‐analysis, synthesised by domain measured. We did not conduct a formal analysis of moderators, or subgroup analyses by age, gender, ethnicity or other factors. However, in synthesising the studies narratively, we include age and gender differences where these were reported. Differences by measures were also assessed, synthesised and reported. For the narrative synthesis, we reported individual findings of studies, reporting change in means and confidence intervals, with *p* values where available, and if not available or appropriate, report other statistics found in the study, such as percentage change. As included studies varied in their choice and reporting of measures, the statistics reported in our narrative synthesis are also inconsistent.

## Results

### Summary of included studies

**Figure 1:**
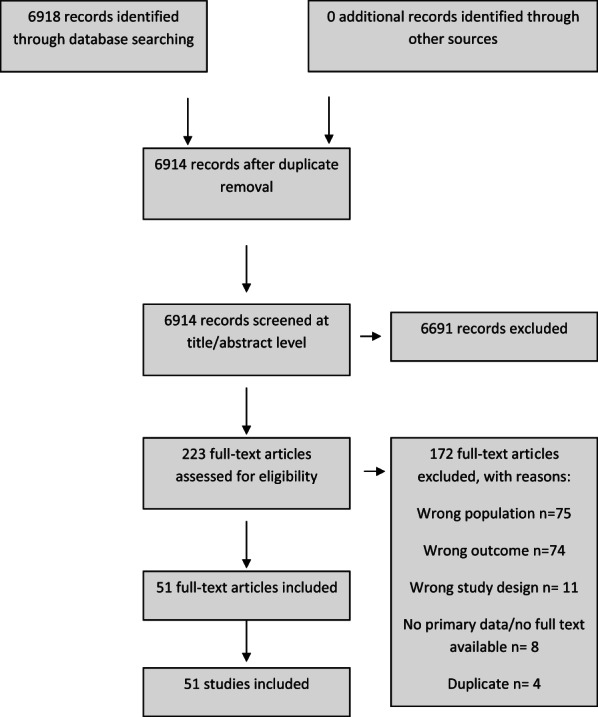
PRISMA diagram

Figure 1 shows a summary of the selection of studies meeting inclusion criteria. In original searches in October 2021, 4,930 records were identified. Our update searches in February 2022 identified a further 1,988 records. In total, 6,917 records were identified. Following deduplication, 6,914 studies were screened at the title and abstract stage, with 6,691 excluded (the majority due to lack of prepandemic data). 223 studies were then assessed for eligibility at full text stage with 172 excluded (75 due to study population, 11 due to study design, 74 due to outcome measures, 4 which were duplicates and 8 having no primary data or full text available). This resulted in a total of 51 studies included in the current review. Characteristics of included studies are shown in Table 1. The findings are discussed by domain of mental health in the sections below. The key findings from individual studies are described in Table S2.

### Study quality and risk of bias in included studies

Only four studies had an overall rating of high quality. Ten were rated as medium/high quality, 17 were medium, seven low/medium and 12 were low quality. In terms of risk of bias assessment (see Figure S1), the most common domains where studies were rated as being at high or unclear risk of bias were on the representativeness of the sample, which was often poorly described, and on strategies to address loss to follow‐up. Eight studies had sampling procedures that were judged to be more likely to be representative of the wider population (Halldorsdottir et al., 2021; Hanno, Cuartas, Miratrix, Jones, & Lesaux, 2021; Hu & Qian, 2021; Khoury, Kaur, & Gonzalez, 2021; Wright, Hill, Sharp, & Pickles, 2021) or school population (Burdzovic Andreas & Brunborg, 2021; Hafstad, Saetren, Wentzel‐Larsen, & Augusti, 2021; Luijten et al., 2021).

### Studies assessing impacts of Covid‐19 on anxiety

#### Study characteristics

Twenty‐one included studies assessed change in anxiety. Five were conducted in Canada, three in the United Kingdom, two from China and two from the United States. One study each was included from Israel, Spain, Australia, Switzerland and the Netherlands. Participants were aged 11–16 at baseline.

Eleven studies used cohort designs; seven used cross‐sectional surveys with follow‐ups and three were comparisons of samples from cross‐sectional or cohort studies. Sample sizes ranged from 96 to 2,099. Five were rated as high or medium/high quality, seven were rated as medium quality and nine rated as low or medium/low quality. Only three studies used probability sampling, the remaining studies were either convenience samples or did not clearly describe their sampling approach.

Most included studies used broad measures of anxiety, which were child‐reported. The Generalised Anxiety Disorder‐7 (GAD‐7; Spitzer, Kroenke, Williams, & Löwe, 2006) was the most used measure, employed in five studies. A range of other broad measures were used, such as the Brief Spence Children's Anxiety Scale (BSCAS; Reardon, Spence, Hesse, Shakir, & Creswell, 2018), Zung Self‐Rating Anxiety Scale (ZSRAS; Zung, 1971) and the Multidimensional Anxiety Scale for Children (MASC; March, Parker, Sullivan, Stallings, & Conners, 1997). Time range between baseline and follow‐up varied between 3 months and almost 3 years but was 1 year or less in most of the studies.

#### Dimensional measures of anxiety: meta‐analysis

Six studies were included in two meta‐analyses.

**Figure 2:**
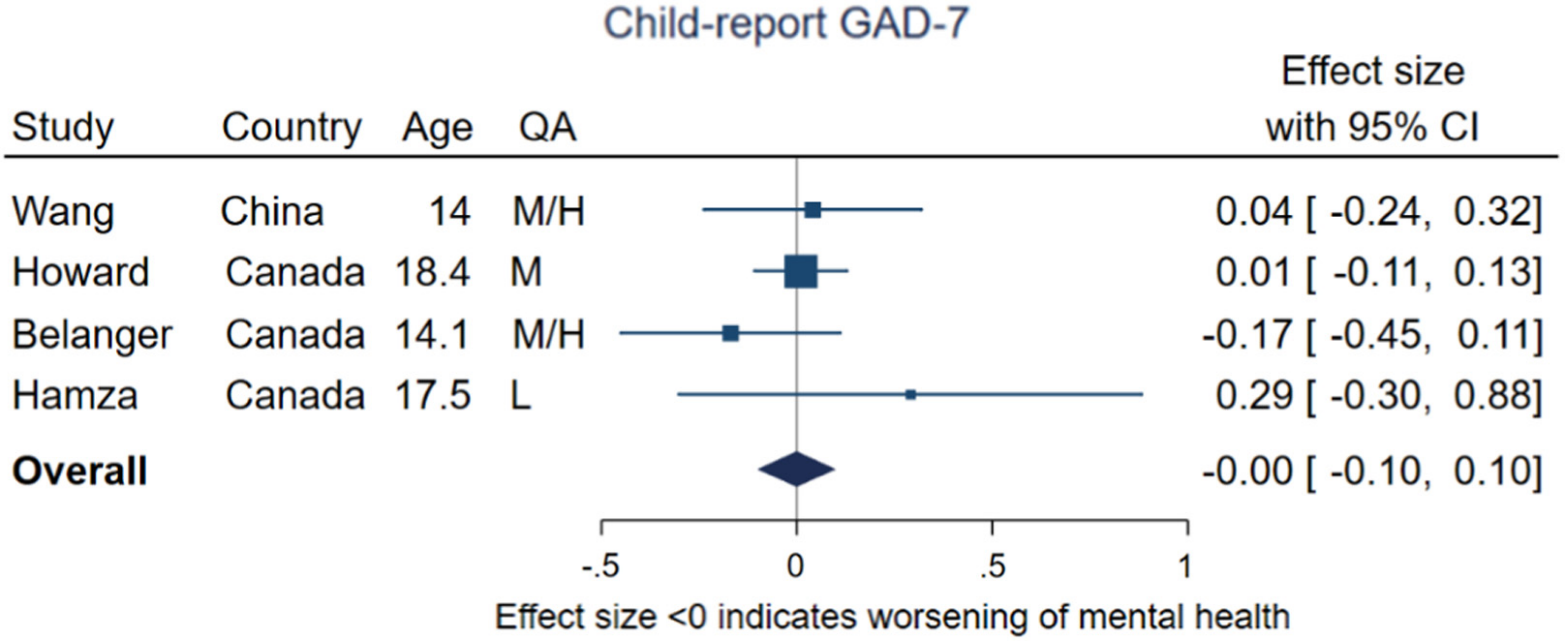
Meta‐analysis of studies using child‐report GAD‐7

Four studies followed the same participants over time and had child‐reported GAD‐7 scores both pre‐ and during‐pandemic (Bélanger, Patte, Leatherdale, Gansaonré, & Haddad, 2021; Hamza, Ewing, Heath, & Goldstein, 2021; Howard, Carnrite, & Barker, 2022; Wang et al., 2021), with a mean age range of 14–18. Three were from Canada, and one from China. The pooled effect size was −0.001 (95% CI −0.10, 0.10, *p* = .98, *I* ^2^ = 0%), indicating no evidence of change in scores (shown in Figure 2). Two of these studies were rated as medium‐high quality, one as medium and one as low. All four studies took place under medium/high or high levels of restrictions, with follow‐up periods from 7 months to 2 years.

**Figure 3:**
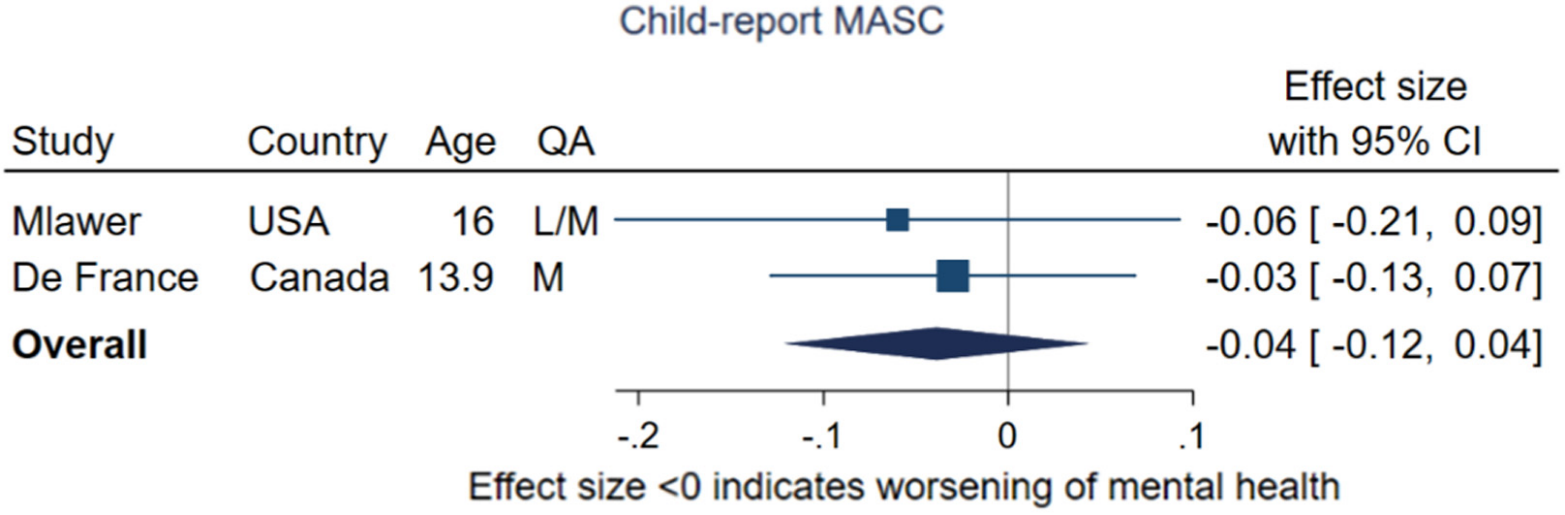
Meta‐analysis of studies using child‐report MASC

The second meta‐analysis of two studies (Figure 3), rated as low or low/medium quality, following adolescents over time using the child‐reported MASC also found little evidence of change from pre‐ to during‐pandemic (pooled effect size −0.04; 95% CI −0.12, 0.04, *p* = .75, *I* ^2^ = 0%). Both collected follow‐up data under conditions of high restriction stringency, but follow‐up periods differed (41–42 months for De France et al. (2021) and <1 year for Mlawer et al. (2022)).

#### Measures of anxiety: narrative synthesis

Seventeen studies included broad measures of anxiety or of generalised anxiety disorder. All except two used samples of adolescents as opposed to younger children. The individual findings from this group of studies were mixed. Four studies, from Australia, Israel, the United States and the Netherlands reported overall increases from prepandemic to within‐pandemic (Luijten et al., 2021; Magson et al., 2021; Mlawer, Moore, Hubbard, & Meehan, 2022; Shoshani & Kor, 2022). These studies collected follow‐up data under conditions of high restriction stringency and included adolescents. One of these was rated high quality (Luijten et al., 2021), with the rest being of medium or low quality. Luijten et al. (2021) used a two‐step random stratified sampling approach, intended to be representative of the Dutch general population, and reported a mean increase of 7.1 (95% CI 6.2–7.9, *p* < .01) points on the Patient‐Reported Outcomes Measurement Information System (PROMIS; Irwin et al., 2010) anxiety domain T‐score. One study from Canada (Hollenstein, Colasante, & Lougheed, 2021) reported a decrease in anxiety scores on the Beck Anxiety Inventory in adolescents (BAI; Steer & Beck, 1997) of −0.19 (95% CI −0.09 to −0.29, *p* = .001). The remainder reported little evidence of change in anxiety scores.

Several studies used subscales or specific measures. One study (Lane et al., 2022) conducted a comparison of cross‐sectional and cohort samples based in Canada (rated as low quality), using subscales of the Screen for Child Anxiety Related Disorders Revised (SCARED‐R; Muris et al., 1998) including social anxiety, separation anxiety, panic and PTSD. Little evidence of a difference was reported on any of these subscales between prepandemic and pandemic samples. Wright et al. (2021) found a relative increase of 26% (95% CI 12–40) in child‐reported symptoms of post‐traumatic stress disorder, using the Child Trauma Screen (CTS; Lang & Connell, 2017), with a corresponding increase in maternal symptom report. Another Canadian study (Hamza et al., 2021) used a convenience sample of older young people (mean age of 18 at follow‐up) and reported no change in symptoms on the Post‐Traumatic Stress Disorder Checklist (Blevins, Weathers, Davis, Witte, & Domino, 2015).

#### Overall conclusions from studies assessing changes in anxiety

Meta‐analyses of broad measures of anxiety found little evidence of changes from prepandemic to during pandemic. Whilst there was some evidence from high‐quality studies of younger adolescents of an increase in anxiety and/or PTSD symptoms, the findings of individual studies appeared inconsistent, with other no obvious pattern evident by age group, setting or design.

### Studies assessing impacts of Covid‐19 on depression

#### Overall study characteristics

Thirty‐one included studies assessed whether depressive symptoms changed from pre‐ to during‐pandemic. Studies assessed either symptom changes over time or ascertained the proportion meeting diagnostic criteria pre‐ and during‐pandemic. Six studies were from Canada, six from the United States and five from China. Other studies were from Norway (*n* = 3), United Kingdom or England (*n* = 3), Germany (*n* = 1), Iceland (*n* = 2), Australia (*n* = 2), Japan (*n* = 1), Israel (*n* = 1), Switzerland (*n* = 1) and the Netherlands (*n* = 1). All participants in studies measuring depression were over the age of 8 years at baseline, with most of the samples having a mean age of 13–14 years at baseline, and the oldest having a mean age of 18 years prepandemic.

Fourteen studies used a cohort design, eight were cross‐sectional studies with a follow‐up survey, eight were comparisons of different cross‐sectional samples and one was a repeated cross‐sectional study with some baseline participants retained at follow‐up, supplemented by new participants. The vast majority of studies used convenience sampling (*n* = 19), six utilised prospective, random or stratified sampling, and the others were unclear on how they selected and recruited their sample. Three studies (Burdzovic Andreas & Brunborg, 2021; Halldorsdottir et al., 2021; Wright et al., 2021) had sampling procedures that were more likely to represent the general population or school population. Sample size ranged from 184 to 11,774 individuals at baseline, and from 51 (Bignardi et al., 2020) to over 85,000 (von Soest et al., 2022) for those included in at least one form of analysis at follow‐up. Studies had a wide time range from their baseline to follow‐up data collection timepoints, ranging from a minimum of 1 month for some participants (Koenig et al., 2021) to up to 60 months for others (Westrupp et al., 2021).

Measures included the Beck Depression Inventory (BDI; Beck, Steer, & Carbin, 1988), the Brief Symptom Inventory Depression subscale (Franke et al., 2017), the Children's Depression Inventory (CDI; Kovacs, 1985), the 10‐ and 20‐item versions of the CES‐D, the Hospital Anxiety and Depression Scale (HADS; Zigmond & Snaith, 1983), the Hopkins Symptom Checklist Depressive Mood Inventory (Lipman, 1986), the PHQ‐8, 9 or PHQ‐Adolescent version (Kroenke et al., 2009; Kroenke, Spitzer, & Williams, 2001; Spitzer & Johnson, 1995), the PROMIS depression scale (Irwin et al., 2010), the Symptom Checklist‐90 (SCL‐90; Derogatis & Unger, 2010) and the Short Mood and Feelings Questionnaire (SMFQ; Angold, Costello, Messer, & Pickles, 1995).

#### Dimensional measures of depressive symptoms: meta‐analysis

Five meta‐analyses were conducted to pool estimates of mean change in symptoms from before to during the pandemic. Of these five meta‐analyses, two found evidence for increases in depressive symptoms, but three found no evidence of change.

**Figure 4:**
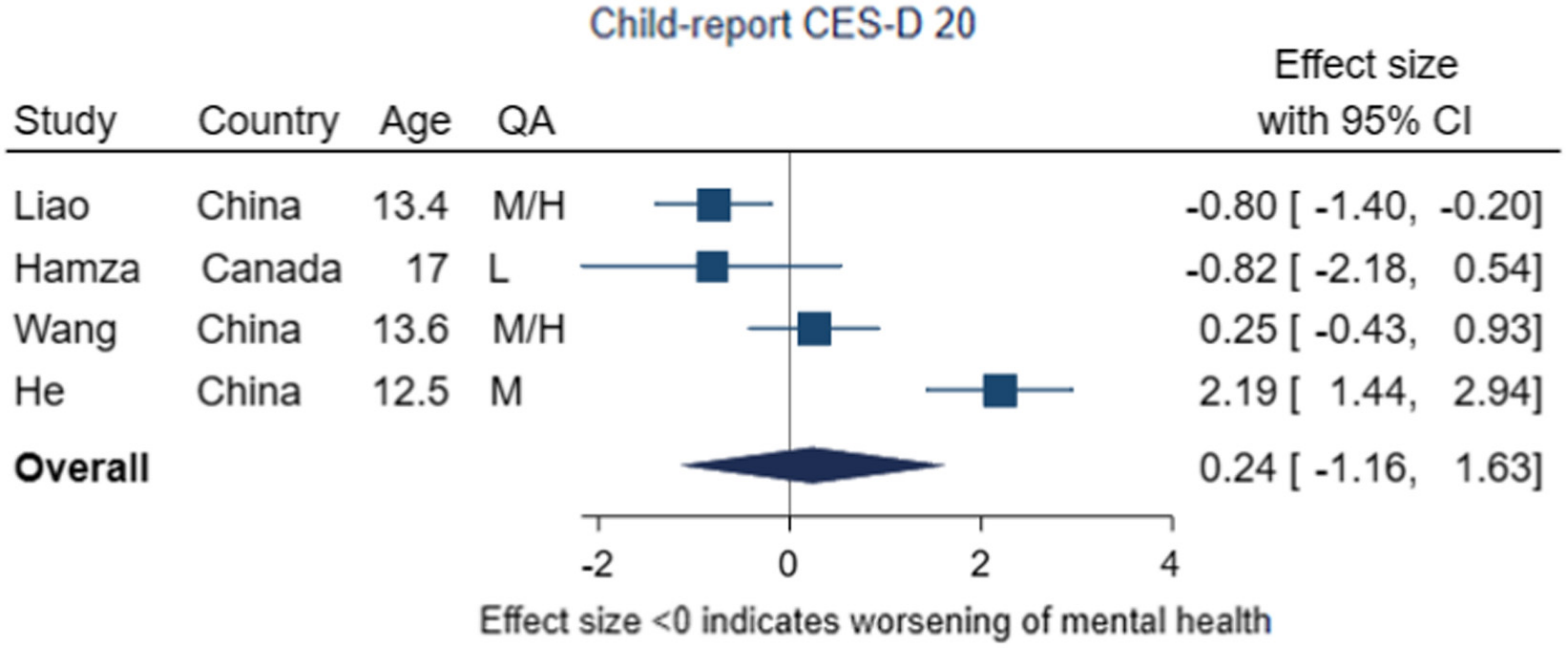
Meta‐analysis of studies using child‐report CES‐D 20

Four studies (Hamza et al., 2021; He et al., 2022; Liao et al., 2021; Wang et al., 2021), all in adolescents, utilised the self‐report CES‐D 20‐item scale, following up the same children over time, with three of the studies set in China and one in Canada (Figure 4). None of the studies were rated as high quality; and only one clearly had a sample representative of the general population (Liao et al., 2021). There was variation in the study results, with one reporting evidence of a decrease in symptoms and another an increase; the pooled effect size was 0.24 (95% CI –1.16, 1.63, *p* = .74), indicating little evidence of change in symptoms of depression from pre‐ to during‐pandemic. The *I* ^2^ statistic was 92%, indicating high heterogeneity across the studies. All studies collected follow‐up data under conditions of medium/high or high restriction stringency.

Two Canadian studies, collecting data under similar levels of restriction stringency assessed child‐reported symptoms using the CES‐D 10‐item scale (Bélanger et al., 2021; Howard et al., 2022). The pooled effect size was −0.65 (95% CI –1.79 to 0.49, *p* = .26) indicating little evidence of changes in symptoms of depression. The studies obtained very different effects, with increasing symptoms of depression in Belanger et al.'s sample of 14‐year olds (rated as medium/high quality), but no evidence of change in Howard et al.'s sample of 18‐year olds (rated as medium quality).

**Figure 5:**
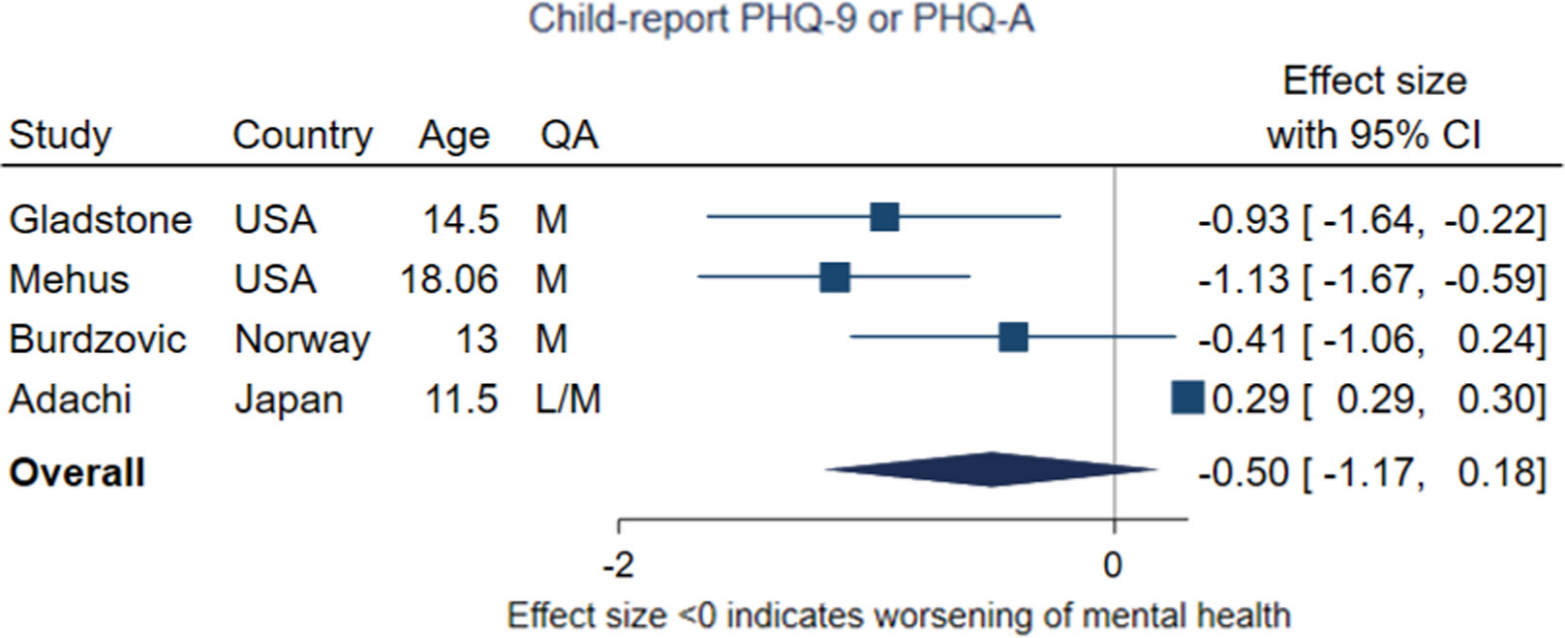
Meta‐analysis of studies using child‐report PHQ‐9 or PHQ‐A

Four studies utilised self‐report measures of the PHQ‐9 or PHQ‐A in the same young people followed over time (Adachi et al., 2022; Burdzovic Andreas & Brunborg, 2021; Gladstone et al., 2021; Mehus et al., 2021) in the United States, Norway and Japan (Figure 5). Again, none of these studies were rated as high quality, although one used a more robust stratified sample of high school students across Norway (Burdzovic Andreas & Brunborg, 2021). The pooled effect from this meta‐analysis was −.05 (95% CI –1.17, 0.18, *p* = .15), with the *p*‐value indicating little evidence of change in symptoms of depression in these samples. Heterogeneity across estimates was again high (*I* ^2^ = 89%). One study (Adachi et al., 2022) found improvements in symptoms whereas the others did not. There were differences in restriction stringency at the time of data collection between the studies in this meta‐analysis, with the lowest levels in Adachi et al. (2022), set in Japan, and higher levels in the two studies from the United States (Gladstone et al., 2021; Mehus et al., 2021). The mean age of participants varied from 11.5 to 18 years.

**Figure 6:**
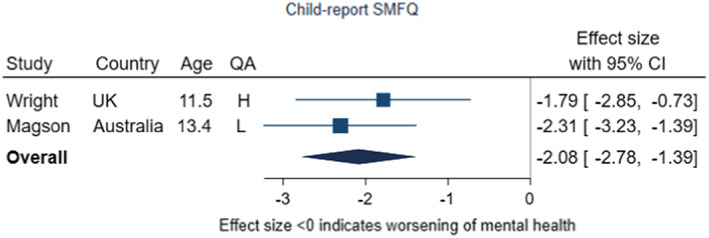
Meta‐analysis of studies using child‐report SMFQ

Two studies examined self‐reported SMFQ scores in samples of younger adolescents followed over time, shown in Figure 6 (Magson et al., 2021; Wright et al., 2021). The pooled effect was –2.08 (95% CI –2.78, −1.39, *p* < .001; *I* ^2^ = 0%). This indicates that from pre‐ to during‐pandemic, scores increased by around two points on the SMFQ, which is scored from 0 to 26 (a deterioration). Wright et al. (2021) was one of the few higher‐quality studies we found, based on a population‐representative birth cohort of pregnant mothers accessing universal antenatal care. Magson et al.'s study was assessed as being low quality. Both had younger samples with ages of 11.5 and 13.4 at baseline and collected data under similar high levels of restriction stringency, despite being from different countries (United Kingdom and Australia).

**Figure 7:**
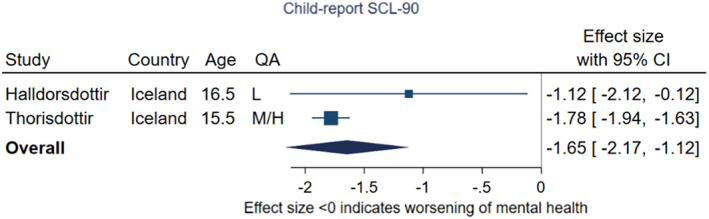
Meta‐analysis of studies using child‐report SCL‐90

Two further studies, one low quality and one medium/high quality, both conducted in Iceland, assessed the change in SCL‐90 scores from pre‐ to during‐pandemic with different samples at baseline and follow‐up (Halldorsdottir et al., 2021; Thorisdottir et al., 2021). The meta‐analysis indicated a pooled effect of –1.65 (95% CI –2.17, −1.12, *p* < .001, *I* ^2^ = 39%), suggesting symptoms of depression increased from the prepandemic sample to the during‐pandemic sample (Figure 7). Both studies collected follow‐up data under conditions of low or low/medium restriction stringency, the mean age of participants was 15–16 years.

#### Measures of depressive symptoms: narrative synthesis

Nine studies that were not included in the meta‐analysis reported increases in symptoms of depression from prepandemic to during the pandemic (Bignardi et al., 2020; De France, Hancock, Stack, Serbin, & Hollenstein, 2021; Ertanir, Kassis, & Garrote, 2021; Gadassi Polack et al., 2021; Hollenstein et al., 2021; Luijten et al., 2021; Mlawer et al., 2022; Shoshani & Kor, 2022; Zhang et al., 2020). Three of these studies included younger children (under 11 at baseline); the rest included predominantly younger adolescents. Only one was rated as high quality (Luijten et al., 2021), using two‐step stratified sampling to achieve a population‐representative sample, reporting an increase in depressive symptoms of 4.9 points (95% CI 4.0–5.7) on the PROMIS depression domain. Overall, these studies were from a range of countries using a range of measures of symptoms of depression. All except one (Ertanir et al., 2021) collected follow‐up data under conditions or high or very high restriction stringency.

Several studies reported wide variability in depression symptom changes across the individuals in their studies. Six studies reported higher increases in females than males (Ertanir et al., 2021; Gladstone et al., 2021; Halldorsdottir et al., 2021; Hollenstein et al., 2021; Magson et al., 2021; Thorisdottir et al., 2021), but other studies found no gender differences. For most of these studies, symptoms increased by a mean of one point from baseline to follow‐up, indicating small clinical changes in symptoms (e.g. one new symptom occurring or one symptom being rated as occurring more often than previously).

Four studies not included in the meta‐analyses found little evidence of a change in symptoms of depression from baseline to follow‐up (Jolliff, Zhao, Eickhoff, & Moreno, 2021; Li et al., 2021; Myhr, Naper, Samarawickrema, & Vesterbekkmo, 2021; Walters, Runell, & Kremser, 2021). Four studies reported no change (Lane et al., 2022; Wang et al., 2021; Westrupp et al., 2021; Widnall et al., 2021). Of these studies, only two were rated as medium/high quality, and only Brunborg (2021) had a sample representative of school students. Other studies were based in the United Kingdom, United States, China and Canada, and there was a mix of designs with several comparing different samples at baseline and follow‐up, and others following up the same children over time. Samples predominantly included adolescents rather than younger children. Restriction stringency at the time of pandemic data collection in these studies was varied.

Five studies compared the proportions of participants meeting threshold cut‐offs on depression scales between pre‐ and during‐pandemic data collection (Burdzovic Andreas & Brunborg, 2021; Li et al., 2021; Myhr et al., 2021; Wang et al., 2021; Zhang et al., 2020). None of these were rated as high quality. Three studies (Burdzovic Andreas & Brunborg, 2021; Myhr et al., 2021; Wang et al., 2021) found little evidence of a change in the proportion of young people meeting threshold criteria, whereas two studies conducted in China found opposing effects: one found a decrease (Li et al., 2021) and another an increase (Zhang et al., 2020). Li et al. (2021) reported a decrease in those meeting diagnostic criteria on the BDI from 35% to 28% over the 3–6 months from baseline to follow‐up (*p* < .001) in sample aged 16 years at baseline, but Zhang et al. (2020) found an increase in a younger sample (12.6 years at baseline) from 18.5% to 24.9% meeting diagnostic cut‐offs on the SMFQ over 6 months (*p* = .01).

This, alongside the findings from the meta‐analyses, suggests that younger adolescents may have been more detrimentally impacted by the pandemic. However, across the individual studies assessing depression there were no clear trends of findings by mean age of participants. In terms of different groups in the population, six of the nine studies found that girls were more negatively impacted than boys.

#### Overall conclusions from studies assessing changes in depression

Of the 31 studies assessing changes in depression, none included children under the age of 8 years and only one study (Bignardi et al., 2020) assessed depressive symptoms in children under 11 years old. Findings were mixed, with no obvious pattern by study quality, study design or setting, except that both high‐quality studies in this section reported increases in depression over time. Two of the five meta‐analyses, and 10 of the 19 other studies found increases in symptoms of depression, with the remainder finding no change, little evidence of change, or even a decrease.

### Studies assessing impacts of Covid‐19 on combined internalising symptoms

Thirteen included studies assessed the change in internalising symptoms. The studies represented a broad range of countries: two each from the United States, Canada, United Kingdom and Germany and one each from Lithuania, Spain, China, Italy and the Netherlands. There was also a wide age range, with age at baseline ranging from 4 to 17 years.

Eleven of the included studies were cohort studies, one of which used a matched samples design, with participants matched on age, sex and type of school. The remaining two were cross‐sectional with follow‐up design. The majority used convenience sampling, with many recruiting subsamples from previous cohort studies. Four (Hu & Qian, 2021; Khoury et al., 2021; Luijten et al., 2021; Wright et al., 2021) used sampling strategies that were more likely to be population or school population‐representative. The sample sizes in included studies ranged from 50 to 1,585. The studies included three rated as medium/high quality, with others assessed as medium or low quality.

Seven studies used the SDQ emotional problems subscale, four of which were parent reported, while the rest were child report. The remaining studies used measures including the Behavioural Assessment System for Children internalising subscale (BASC; Reynolds, 2010), GAD‐7, Child Behaviour Checklist Brief Problem Monitor internalising subscale (CBCL‐BPM; Pedersen et al., 2021) and the Revised Child Depression and Anxiety Scale (internalising) scores (RCADS; Chorpita, Yim, Moffitt, Umemoto, & Francis, 2000).

#### Dimensional measures of internalising symptoms: meta‐analysis

**Figure 8:**
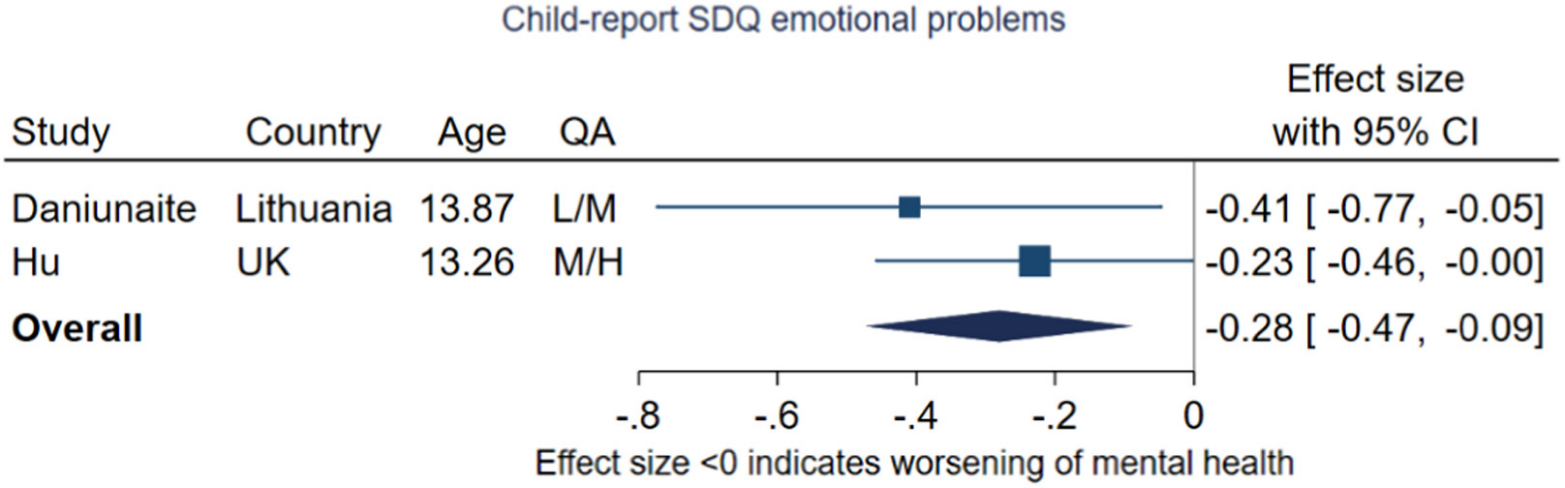
Meta‐analysis of studies using child‐report SDQ emotional problems subscale

Two studies were included in a meta‐analysis to pool estimates of change on the child‐report SDQ emotional problems subscale, shown in Figure 8 (Daniunaite, Truskauskaite‐Kuneviciene, Thoresen, Zelviene, & Kazlauskas, 2021; Hu & Qian, 2021). The pooled effect size was −0.28 (95% CI −0.47, −0.09, *p* = .004, *I* ^2^ = 0%) with the point estimate equivalent to an increase of 0.3 points on the raw emotional problems subscale (with possible range 0–10). Both included adolescents with a mean age of 13 years. Hu and Qian (2021) used the UK Understanding Society cohort (which uses probability sampling) and reported a smaller effect size than Daniunaite et al. (2021). The first of these studies took place under high restriction stringency, and the second under low to medium restriction stringency.

**Figure 9:**
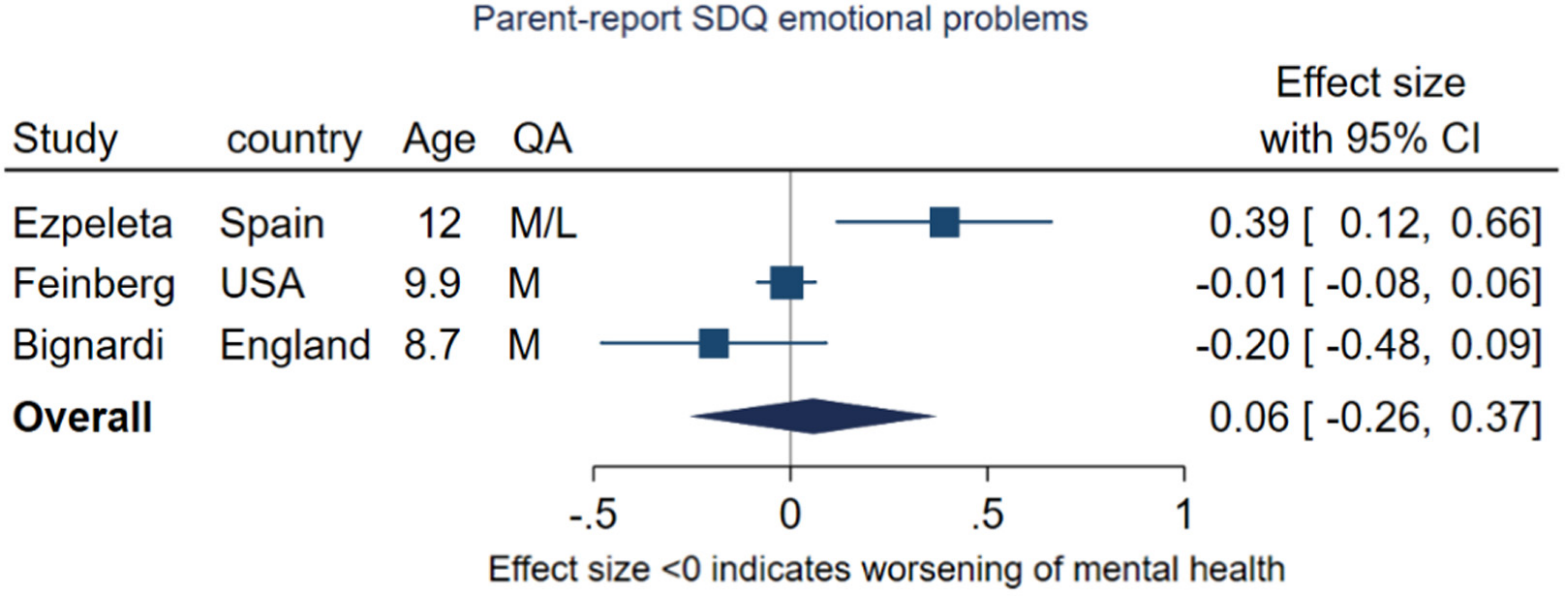
Meta‐analysis of studies using parent‐report SDQ emotional problems subscale

Three studies using the parent‐report SDQ emotional problems subscale were suitable for meta‐analysis (Figure 9; Bignardi et al., 2020; Ezpeleta, de la Osa, Trepat, Navarro, & Penelo, 2020; Feinberg et al., 2021). The pooled effect size was 0.06 (95% CI −0.26, 0.09, *p* = .72), indicating little evidence of change in symptoms from prepandemic to during the pandemic. However, the *I* ^2^ value (85.3%) indicates substantial heterogeneity. Ezpeleta et al.'s (2020) study based in Spain (rated as low‐quality) reported a decrease in parent‐reported emotional problems, contrasting with Feinberg et al.'s (2021) US study, which found no change, and Bignardi et al.'s (2020) UK study which found little evidence of change. These three studies included children with a mean age of 12, 9.9 and 8.7 years respectively. The UK and US studies collected data under conditions of high restriction stringency, and the Spanish study under medium stringency.

#### Internalising symptoms: narrative synthesis

Five of the eight studies not included in the meta‐analyses reported an increase in internalising symptoms in children (Frigerio, Nettuno, & Nazzari, 2022; Hanno et al., 2021; Hu & Qian, 2021; Khoury et al., 2021; Ravens‐Sieberer et al., 2022); this included both parent‐report and child‐reported measures. These studies were based in the United States, United Kingdom, Italy, Germany and Canada, respectively, and all collected data under conditions of high/very high restriction stringency, with the exception of Ravens‐Sieberer et al. (2022). Three studies of older adolescents (mean age of samples 14 years or higher) also found no change or difference in scores of internalising symptoms (Hamza et al., 2021; Koenig et al., 2021; van der Laan et al., 2021) based in Canada, Germany, and the Netherlands and with restriction stringency being high or medium. There were no apparent differences in the design or quality of studies between those that found evidence of change compared to those who found no difference. Participants in the three studies that reported no change in symptoms were on average older than those reporting a worsening of internalising symptoms. All included studies reported on gender, with four reporting that girls on average experienced a greater increase in internalising symptoms (Daniunaite et al., 2021; Ravens‐Sieberer et al., 2022; van der Laan et al., 2021).

#### Overall conclusions from studies assessing changes in internalising symptoms

Meta‐analysis based on child‐reported internalising symptoms found evidence of an increase in these difficulties. In contrast, meta‐analysis of parent‐reported internalising symptoms, in a younger age group, found no evidence of change. Again, findings from the heterogenous group of individual studies were mixed but indicated that younger children may be more likely to have experienced a deterioration in internalising symptoms.

### Studies assessing impacts of Covid‐19 on externalising symptoms

#### Overall study characteristics

Eleven included studies assessed change in externalising behaviours, with the majority reporting change in mean scores (Daniunaite et al., 2021; Ezpeleta et al., 2020; Feinberg et al., 2021; Frigerio et al., 2022; Hanno et al., 2021; Hu & Qian, 2021; Khoury et al., 2021; Koenig et al., 2021; Ravens‐Sieberer et al., 2022; Walters et al., 2021; Wright et al., 2021). Three included studies were carried out in Germany and two in the United States and United Kingdom, with one study each set in Lithuania, Spain, Canada and Italy. The age profile at baseline of these studies was slightly younger than those on internalising symptoms, ranging from 4 to 15 years at baseline.

Six studies used a cohort design, with other designs being cross‐sectional with follow‐up (*n* = 2), cross‐cohort or cross‐sectional comparison (*n* = 2) and within‐cohort matched design (*n* = 1). Sample size at follow‐up ranged from 59 to 1,585, with follow‐up periods from 1 to 48 months postbaseline. Three (Hu & Qian, 2021; Khoury et al., 2021; Wright et al., 2021) used sampling strategies designed to be population‐representative, with the remainder being convenience samples. This section included one high quality study, and three medium/high quality studies. Whilst four studies investigated general externalising symptoms; others measured specific dimensions including hyperactivity/inattention, attention problems, conduct problems, dysregulation, and impulsivity. The SDQ subscales were used in six studies; other measures used included the BASC, the CBCL and the Weinberger Adjustment Inventory (WAI) impulse control scale (Weinberger, 1997).

#### Dimensional measures of externalising problems: meta‐analysis

**Figure 10:**
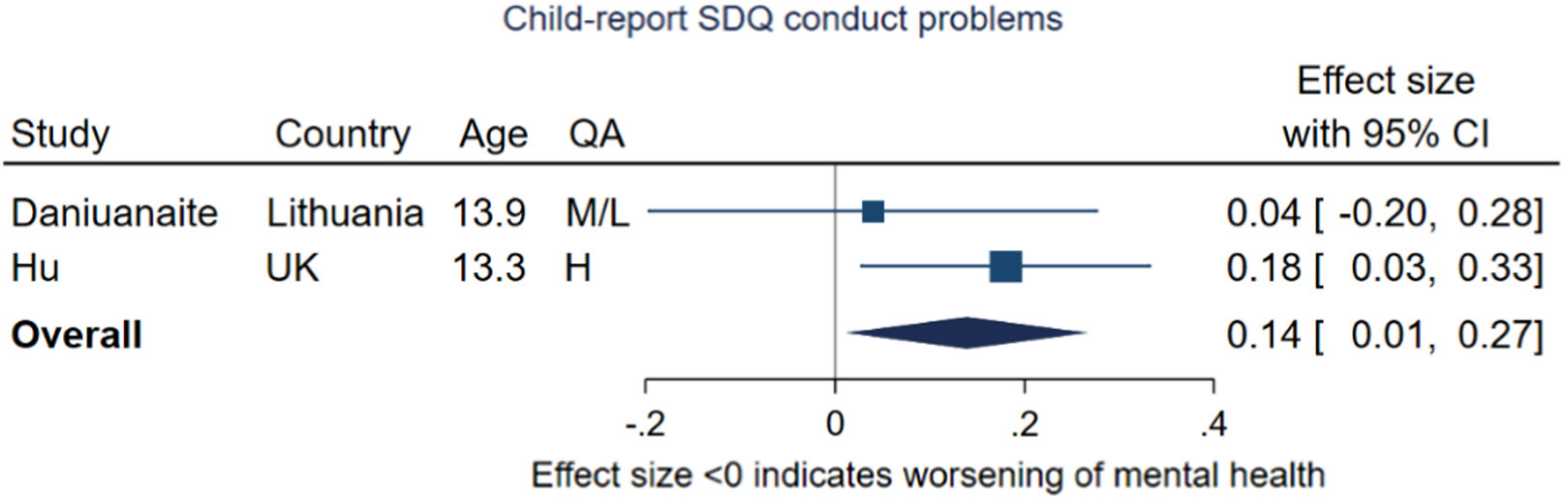
Meta‐analysis of studies using child‐report SDQ conduct problems subscale

Two studies contributed to the two meta‐analyses of externalising problems, as they both reported changes on the SDQ subscales for conduct problems and for hyperactivity/inattention, reported by the child using study designs following the same children over time (Daniunaite et al., 2021; Hu & Qian, 2021). For conduct problems, the pooled effect size was 0.14 (95% CI 0.01, 0.27, *p* = .03, *I* ^2^ = 0%), indicating evidence of a decrease from pre‐ to during‐pandemic of equivalent to approximately 0.14 points on the raw ten‐point scale (Figure 10). These two studies included participants of similar ages (mean age of 13 years) but differed in their findings. Hu and Qian (2021) reported a decrease in conduct problems in their UK sample under high restriction stringency, and Daniunaite et al. found no evidence of change in their school sample under low/medium stringency.

**Figure 11:**
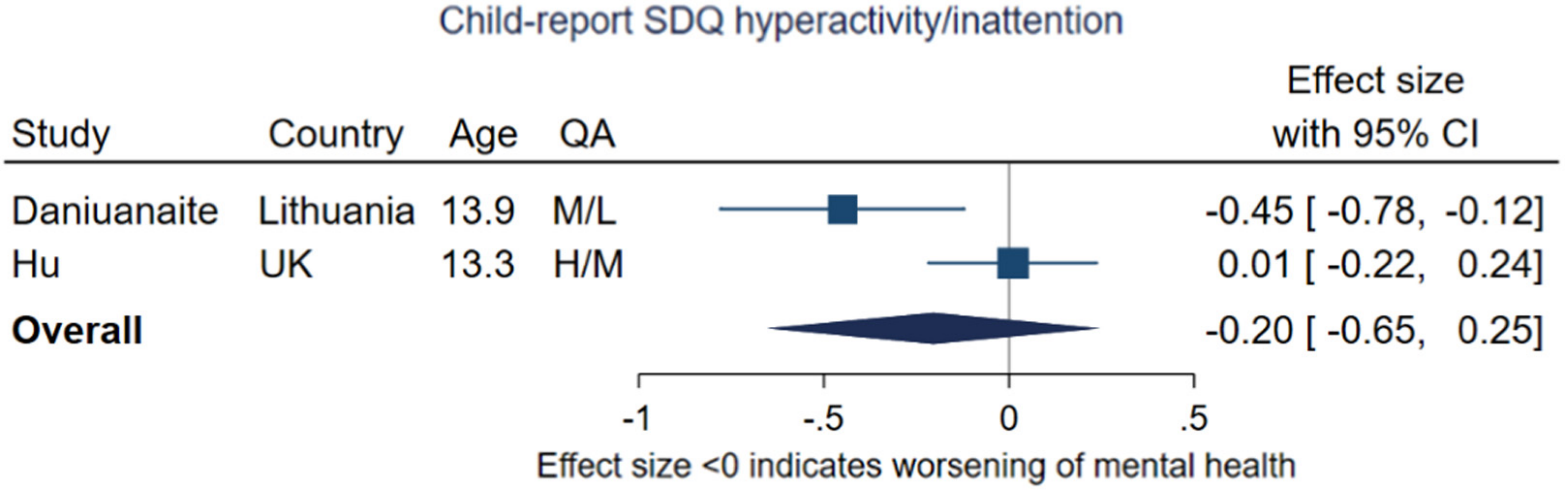
Meta‐analysis of studies using child‐report SDQ hyperactivity/inattention problems subscale

There was little evidence for a change in hyperactivity and inattention, based on the pooled effect size (−0.20, 95% CI −0.65, 0.25, *p* = .37), shown in Figure 11. The two included studies again had dissimilar estimates (*I* ^2^ indicated high heterogeneity at 80.4%) with Daniuanaite et al. reporting an increase in symptoms, whereas Hu and Qian found no difference in symptoms from pre‐ to during‐pandemic.

#### Measures of externalizing problems: narrative synthesis

Nine studies did not contribute to the meta‐analysis. Feinberg et al. (2021) reported on externalising behaviours using the SDQ in a sample with a mean age of 9.9 years in the United States. The authors found evidence of an increase in those in the clinical range from 12.1% to 16.5% (*p* = .05), which was correlated with lockdown. Another study from the United States (Hanno et al., 2021) used the BASC in children with a mean age of four at baseline; this was rated as medium‐high quality. The authors reported fixed effects analyses showing children's externalizing and dysregulated behaviours increased during the pandemic, with a change of approximately 0.2 standard deviation units (0.11 points; 95% CI: 0.06–0.16). Both US studies were conducted under similar levels of relatively high restriction stringency. A third Canadian study (Khoury et al., 2021), also in younger children using a smaller sample from a cohort study (sociodemographic characteristics of the sample did not differ from the larger population representative cohort), found evidence of an increase in externalising behaviours on the CBCL Brief Problem Monitor (mean difference of 4.35 on *T* score, *p* < .001). Wright et al. (2021) used the CBCL aggression subscale and found evidence for an increase of 76% in mother‐reported externalising problems. These two studies also took place under conditions of high restriction stringency.

Three further studies examined changes in conduct problems. Two studies set in Germany, during similar levels of restriction stringency (medium) and with similar age groups, reported differing results. Ravens‐Sieberer et al. (2022) reported an increase in the proportion of participants scoring above threshold in the conduct disorders subscale of the SDQ in their cross‐cohort study from 6.6% to 10% (*p* < .01) with a representative population sample. In contrast, Koenig et al. (2021), using a convenience sample, found a mean decrease of 0.16 points (95% CI: −0.32, −0.02, *p* = .026). A further cohort study set in Spain reported an increase but was rated as medium/low quality (Ezpeleta et al., 2020).

Three studies not included in the meta‐analysis used the SDQ hyperactivity/inattention subscale. Ravens‐Sieberer et al. reported higher levels of hyperactivity/inattention in their pandemic sample than their prepandemic participants (14.6% above the subscale cut‐off compared to 7.7%, *p* < .001). Two reported no change (Ezpeleta et al., 2020; Koenig et al., 2021). Studies using other measures included a very small birth cohort study in Italy, collecting data in a period of high or very high restriction stringency, including 59 children with a mean age of 4–5 years during the pandemic (Frigerio et al., 2022). Their trajectory analysis showed an increase in attention problems on the CBCL attention problems subscale (*p* = .001), although the study was rated low quality. Another low‐quality study from the United States, also under high restriction stringency (Walters et al., 2021) reported an increase in impulsivity on the Weinberger Adjustment Inventory Impulse Control scale over a 12‐month period (*p* = .006), in a sample with a mean age of 12 years. Several studies reported examining effects of gender (Hu & Qian, 2021; Khoury et al., 2021; Ravens‐Sieberer et al., 2022; Wright et al., 2021) but only one reported finding any effect, finding a greater increase in externalising symptoms in boys (Frigerio et al., 2022).

#### Overall conclusions from studies assessing changes in externalising problems

The two meta‐analyses found an improvement in conduct scores, but little evidence for change in hyperactivity/inattention, although each only included two studies. Findings from individual studies in the United States and Canada, all rated at least medium quality, as well as one high‐quality UK‐based study appeared to support an increase in externalising problems overall; it may be worth noting that restriction stringency was relatively high in these settings. Individual studies examining conduct and hyperactivity/impulsivity dimensions had more mixed results and included lower quality studies. There were, otherwise, no discernible pattern in the findings, although these studies were mostly in younger children rather than adolescents.

### Studies assessing impacts of Covid‐19 on prosocial or peer relationship problems

#### Study characteristics

Four studies reported changes in prosocial skills and/or peer relationship problems, all using the subscales from the SDQ (Daniunaite et al., 2021; Ezpeleta et al., 2020; Hu & Qian, 2021; Koenig et al., 2021). These studies were set in Lithuania, Spain, United Kingdom and Germany respectively, and included an age range from 10 to 20 years. Three were of cohort design, with one being a within‐cohort comparison. Only one was of medium/high quality and used a probability sample (Hu & Qian, 2021). Timing of follow‐up varied from 4 months to 2 years.

#### Measures of peer relationship problems and prosocial behaviour: meta‐analysis

Due to the variation described above, it was only possible to conduct two meta‐analyses. These included the same two studies (Daniunaite et al., 2021; Hu & Qian, 2021). Hu and Qian's study, set in the United Kingdom took place under relatively high levels of restriction stringency and utilised a robust sampling strategy, whereas Daniuaite et al. collected data in Lithuania under lower levels of restrictions. The effect sizes of the two included studies were very different, with only the UK study finding evidence of an increase in peer relationship problems. Both studies included participants with a mean age of 13 years.

**Figure 12:**
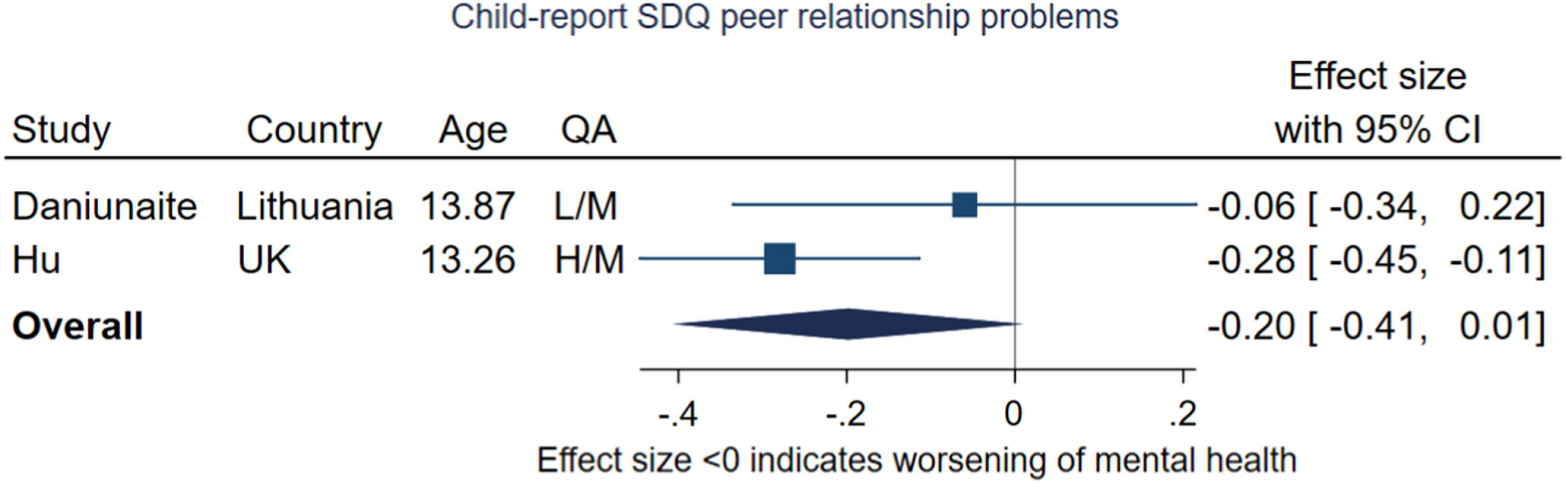
Meta‐analysis of studies using child‐report SDQ peer relationship problems subscale

On meta‐analysis, there was tentative evidence of an increase in peer relationship problems from prepandemic to within pandemic (Figure 12); pooled effect size was −0.20 (95% CI −0.41, 0.01, *p* = .06, *I* ^2^ = 44.5%).

**Figure 13:**
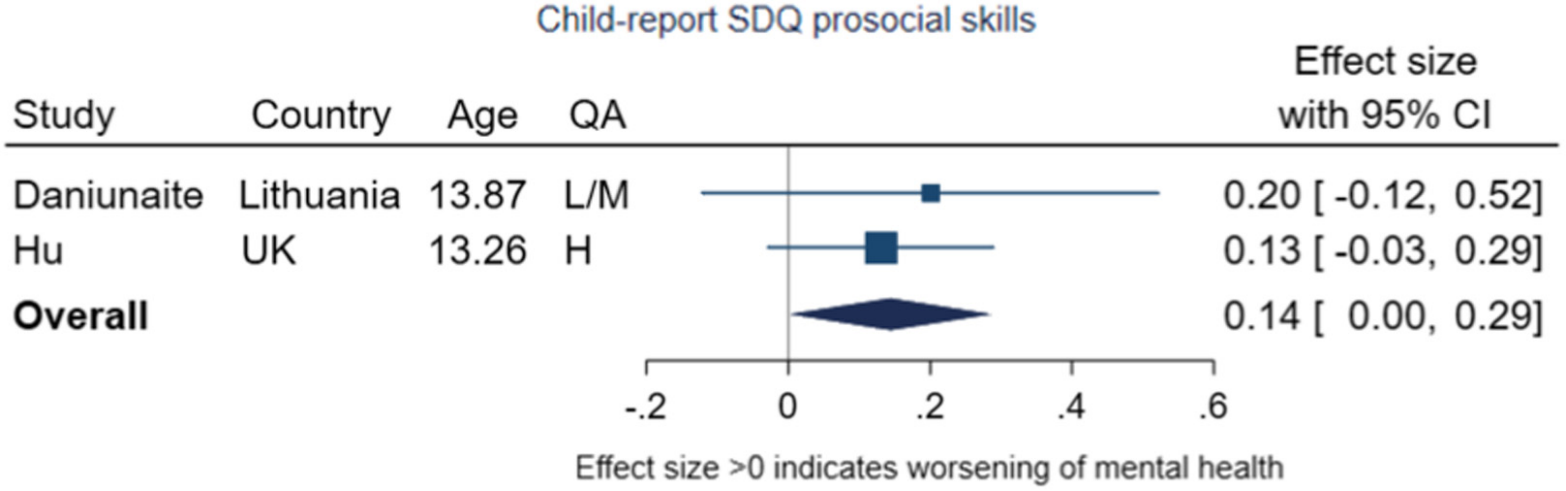
Meta‐analysis of studies using child‐report SDQ prosocial skills subscale

The pooled effect size for changes in prosocial behaviour was 0.14 (95% CI −0.00, 0.29, *p* = .05, *I* ^2^ = 0%), providing weak evidence of a deterioration in prosocial behaviour from pre‐ to during‐pandemic (Figure 13).

#### Measures of prosocial behaviour and peer relationship problems: narrative synthesis

Two studies measuring change in prosocial behaviour and peer relationship problems (on the SDQ) were not included in the meta‐analysis. Koenig et al.'s (2021) study set in Germany, rated medium‐high quality, reported no difference in child‐rated prosocial or peer relationship problem scores between prepandemic and pandemic groups in a sample with a mean age of 15 years. Ezpeleta et al. (2020) reported an increase in parent‐reported prosocial behaviour in children with a mean age of 12 years, in a low‐medium quality cohort study set in Spain (mean change of 1.4, 95% CI 1.13, 1.67, *p* < .001), but also found that peer relationship problems increased (mean change of 0.88, 95% CI: 0.66, 1.09, *p* < .001). Restriction stringency ratings were similar, although in Spain children and young people had experienced restrictive stay‐at‐home orders that had not long been relaxed at the time of data collection. There was no clear pattern in findings in terms of effects of gender; two studies did not clearly report gender effects (Ezpeleta et al., 2020; Koenig et al., 2021), one study reported a greater decline in prosocial behaviour in boys (Hu & Qian, 2021), and one suggested a more marked increase in prosocial behaviours in boys, influenced by lower prepandemic levels of these behaviours (Daniunaite et al., 2021).

#### Overall conclusions from studies assessing changes in peer relationship problems and prosocial behaviours from pre‐ to during‐pandemic

Of the four studies assessing changes in prosocial behaviours and/or peer relationship problems, none included children under the age of 10 years at baseline. Only one study used probability sampling. Meta‐analysis of two studies including prosocial skills and peer problems measures found weak evidence indicating a slight decrease (or deterioration) in both domains.

### Studies assessing impacts of Covid‐19 on total mental health difficulties or global severity

#### Study characteristics

Six studies reported the change in total mental health difficulties or global severity (Ezpeleta et al., 2020; Hafstad et al., 2021; Hussong, Midgette, Thomas, Coffman, & Cho, 2021; Koenig et al., 2021; Metherell, Ghai, McCormick, Ford, & Orben, 2021; Shoshani & Kor, 2022). Most were set in European countries, including Germany (*n* = 1), Norway (*n* = 1), Spain (*n* = 1) and England/United Kingdom (*n* = 1). One study was set in Israel and another in the United States. There was a wide age range of participants, from 10 to 20 years. Most were of cohort design, with two cross‐sectional studies with follow‐ups, and one within‐cohort comparison. Three studies used probability sampling strategies, two of which used the UK Understanding Society study (Hu & Qian, 2021; Metherell et al., 2021); and Hafstad et al. (2021) utilised a cohort from a probability sample based on stratified sampling of Norwegian schools; the remaining studies appeared to use convenience samples, but the methods were not well described. Three studies were of medium/high quality, one of medium quality, three of medium/low quality and one of low quality according to our appraisal.

Five studies used the SDQ total difficulties score as their measure. Other measures used included the HSCL‐10 self‐report questionnaire (Lipman, 1986), the Paediatric Symptom Checklist (PSC; Jellinek, Murphy, & Burns, 1986), and the Brief Symptom Inventory‐18 (Franke et al., 2017).

Sample sizes ranged from 88 to 3,572 individuals for those included in at least one form of analysis at follow‐up. Studies had a wide range of time from their baseline to follow‐up data collection timepoints, ranging from several months for some participants (Hu & Qian, 2021; Koenig et al., 2021) to more than 70 months for others (Metherell et al., 2021). The lockdown stringency in each setting during the pandemic data collection also varied widely.

#### Measures of total difficulties or global severity: narrative synthesis

Studies measuring total difficulty or global severity were heterogeneous in their characteristics, and it was not possible to conduct meta‐analyses. All except one of the six found evidence of an increase in scores upon follow‐up, although some reported small effect sizes. The exception was Koenig et al.'s study set in Germany, which found lower self‐rated scores in children surveyed during the pandemic than prepandemic, using matched pairs of children from the same cohort (Koenig et al., 2021).

#### Overall conclusions from studies assessing changes in total difficulty or global severity, from pre‐ to during‐pandemic

The six studies assessing changes in total mental health difficulties or global severity included a similar age range of children and adolescents, none included children under the age of 10 years at baseline. The studies were otherwise varied in design, measure, and setting. It was not possible to conduct meta‐analyses of studies reporting total difficulties or global severity, although the pattern of findings from individual studies indicated an increase in scores.

### Studies assessing impacts of Covid‐19 on self‐harm, and suicidal ideation

There were 13 ‘other’ symptoms, disorders or other mental health domains measured across five included studies. These were a diverse range of studies which assessed the change in symptoms, the change in proportion of children experiencing symptoms or the relative rates of events not otherwise specified in the present study, from pre‐ to during‐pandemic. The five studies were conducted in Canada, Germany, England, China and Mexico, and included a wide age range and different designs. The quality of included studies also ranged from high (Odd et al., 2021) to low (Hamza et al., 2021). These studies assessed self‐harm, borderline personality disorder/Emotionally Unstable Personality Disorder, suicidal ideation and suicidal attempts, eating disorders and rates of suicide. The specific measures used were the Adapted version of the Inventory of Statements about Self‐Injury (Klonsky & Olino, 2008), McLean Screening Instrument for Borderline Personality Disorder (MSI‐BPD; Zanarini et al., 2003), Youth Risk Behaviour Surveillance System (Everett, Kann, & McReynolds, 1997) and the Eating Disorder Examination – Questionnaire (EDE‐Q; Fairburn & Beglin, 1994), all of which were self‐reported. None of the studies were similar enough to combine in a meta‐analysis.

One large cohort study set in schools in a province of China used the Youth Risk Behaviour Surveillance System to report on self‐harm and suicidal ideation, plans and attempts (Zhang et al., 2020). Zhang et al. found increases in the proportion of students reporting these thoughts and behaviours over a 6‐month period from prepandemic to during the pandemic (May 2020, during a period of high restriction stringency). The percentage of children who had reported suicide attempts doubled (3.0–6.4%), and nonsuicidal self‐injury increased by 11 percentage points (31–42%).

In contrast, Koenig et al.'s matched‐pair study in Germany (with lower restriction stringency) found a decrease in reported suicide plans in the during‐pandemic period compared to the prepandemic period (*p* = .007) but noted that plans were reported by very few participants in either sample. No difference in scores on the EDE‐Q was found between the pre and during pandemic samples in the same study (Koenig et al., 2021).

Valdez‐Santiago et al. (2022) reported a cross‐cohort comparison of rates of self‐reported suicide attempts in 10‐ to 19‐year olds in Mexico, using data from the Mexican National Health and Nutrition Surveys. Little evidence of a difference in rates was found between the 2018–2019 and 2020 survey waves. Analysis suggested a trend of an increase in suicide attempts in females and a decrease in males, but confidence intervals overlapped.

## Discussion

### Summary of findings

This review found 51 studies meeting our inclusion criteria, which measured change in a range of aspects of mental health from prepandemic to during‐pandemic in children and young people. These studies were diverse in terms of design, setting, timing in relation to the pandemic, population, length of follow‐up and choice of measure. Despite the application of criteria intended to select ‘high‐quality’ studies, included studies varied in quality, and only nine of the 51 studies utilised sampling strategies that could be considered representative of the general population or school population. Reporting of design and basic descriptive statistics were particularly poor in many studies, possibly owing to pressures to publish quickly on this topic.

Methodological heterogeneity limited the potential to conduct meta‐analyses across studies and provide meaningful overall estimates of effect. On balance, however, the evidence is mixed, with some suggestion of a small deterioration across the broader measures of mental health, such as an increase in total difficulty or global severity scores, externalising problems and internalising symptoms.

This is, however, a simplified overview of a diverse and sometimes contradictory body of evidence, which is likely to reflect the quite different experiences of children, young people and families across a range of circumstances and settings. It also reflects the challenges of selecting, combining and summarising this body of research, which required decisions to be made in terms of analysing and presenting these findings in a concise and meaningful way. We discuss some of these nuances in more detail below.

Our findings are in broad agreement with other reviews attempting to synthesise the evidence on this question, which also concluded there had been a deterioration, but with multifaceted effects (Samji et al., 2022; Theberath et al., 2022). Our finding of a relatively slight effect at population level are also in line with one of the only other meta‐analyses published at the time of writing (Bussières et al., 2021). This meta‐analysis included 28 longitudinal studies in children and reported a negative general mental health impact ‘of small magnitude’. However, to our knowledge, our review is the first systematic review to attempt to synthesise evidence on the impact of Covid‐19 across a range of domains of mental health.

Interestingly, the deterioration we found in our review appears less marked than in one of the studies we could not include, as the data had not yet been made available for access by researchers. This was the Mental Health of Children and Young People in England (MHCYP) survey series, which involved follow‐up waves in August 2020 and February/March 2021 to the baseline cross‐sectional survey performed in 2017, using the Strengths and Difficulties Questionnaire algorithm (Goodman, Renfrew, & Mullick, 2000). The survey series used a well characterised probability sample but was affected by attrition over time, using weighting to adjust for this. The MHCYP surveys found an increase in the prevalence of probable disorder in 5‐ to 16‐year olds from 10.8% in 2017 to 16% in 2020, with a significant increase seen across boys and girls. This rise was maintained in the subsequent 2021 survey wave nine months later (Newlove‐Delgado et al., 2021). The timing of data collection in the surveys may have influenced the sustained high prevalence level, with the 2021 survey taking place during England's spring 2021 lockdown, where the stringency index was almost 90/100 (Hale et al., 2021). However, when individual‐level change from 2017 to 2021 was examined, the picture was more mixed, with 39.2% of those aged 6–16 years in 2021 experiencing deterioration in mental health since 2017, and 21.8% experiencing improvement. It is likely to be the case that such cross‐sectional comparisons of prevalence, which are valuable in terms of providing headline figures, fail to capture the nuance and varying trajectories of children over time, which are apparent in this review.

We also reported differences in change over time by type or domain of difficulty. Our meta‐analyses of studies measuring generalised anxiety found little evidence of change. This may reflect the use of broad measures of anxiety, which would not identify changes in different *types* of anxiety during the pandemic. For example, it has been suggested that symptoms of anxiety, and social anxiety in particular, may have decreased for some children, due to a decline in exposure to social interaction or unfamiliar situations or scenarios (Barendse et al., 2022). Timing of measurement may therefore be particularly important against a background of changing levels of social interaction in many settings. A narrative review of studies early in the pandemic (mostly from China) suggested that estimates of anxiety prevalence were highest in the periods shortly after enactment of lockdown measures (Samji et al., 2022). Similarly, in terms of measurement, anxiety symptom scales may have been especially sensitive to context and opportunities for exposure, which would vary during fluctuations in restrictions.

Our findings regarding depression were mixed. We note that studies using categorical measures found less evidence for increases in symptoms than those using dimensional measures, this may reflect the findings that increases in symptoms were small in magnitude. Several high‐quality studies reported an increase in symptoms of depression, and two of the five meta‐analyses found evidence for significant increases in depressive symptoms, yet three meta‐analyses found little evidence of change. Again, it is challenging to fully explain these results. For example, one meta‐analysis included three studies using the child‐report CES‐D in China, in samples with similar ages (He et al., 2022; Liao et al., 2021; Wang et al., 2021). These studies had different estimates of effect, with one reporting an improvement, one finding no evidence of change, and one reporting a deterioration, however the quality of studies was mixed, and samples did not appear to be population‐representative. There was some indication of age and gender effects. Six of nine studies reporting on gender found more marked increases in depressive symptoms in girls, which mirrors patterns in studies of adults suggesting that women were more likely to experience increases in depressive symptoms during the pandemic (Pierce et al., 2020; Vloo et al., 2021). Studies reporting deterioration also generally included younger rather than older adolescents. This may reflect age‐related increases in depression in early adolescence (discussed further below) rather than pandemic‐related change. The overall impacts of pandemic‐related changes to individual, family, social, and environmental factors which may influence depressive symptoms are also likely to be finely balanced. For example, the effect of school closures or social distancing measures may have been neutral or even positive for some young people, by reducing exposure to negative interpersonal relationships and peer victimisation that contribute to risk of depression (Stapinski, Araya, Heron, Montgomery, & Stallard, 2015).

In spite of these mixed findings on measures of depression there was evidence of an increase in broader internalising symptoms based on child report (although with wide confidence intervals), but not parent report. These internalising scales measure both depression and anxiety. We have no clear explanation for this finding; one possibility is that there is a measurement effect, and children asked to reflect on a broader range of internalising symptoms of mental health (e.g. the SDQ) were more likely to perceive a global impact on their mental health and endorse these than those who completed a brief focussed measure about one domain of their mental health (e.g. the PHQ).

On meta‐analysis, we found evidence of improvements in child‐reported conduct problems. This might relate to the differences in the child's environment during restrictions, for example, problems with peers or with other behaviours may be less apparent when a child is not attending school, or where the stressors and demands of in‐person education are removed. However, findings from studies in the United States, United Kingdom and Canada appeared to support an increase in externalising problems overall (Hanno et al., 2021; Khoury et al., 2021; Wright et al., 2021), and studies from a range of settings, largely with higher levels of restriction stringency also reported negative changes in hyperactivity, inattention and impulsivity (Daniunaite et al., 2021; Hu & Qian, 2021; Khoury et al., 2021; Walters et al., 2021). The findings suggested that younger children and boys may have experienced more deterioration on these measures.

Whilst we did not formally conduct subgroup analysis or analyses of moderators, in general, studies in this review indicated a more marked effect on younger children overall. Whilst this may relate to greater developmental vulnerability of younger children, there may be other factors contributing to this finding. The effect of ageing within cohorts can give the impression of increased prevalence, especially in studies with longer periods between prepandemic and pandemic data points, although in general there was no consistent pattern apparent by length of follow‐up in our review. Although some studies adjusted for age, this does not account for the natural development of mental health problems in childhood, where some periods are particularly associated with changes in trajectories. For example, longitudinal studies point to decreases in externalising symptoms after preschool age and increases in depression during early adolescence (Olson et al., 2017; Picoito et al., 2021). This review includes a range of measures, age groups, settings, and follow‐up periods, and for many of the included measures, norms matching these are not available. This makes it challenging to judge the extent to which changes in scores over time represent normal patterns of developmental change. We therefore suggest that the findings (and further research) are considered with this caveat in mind.

Studies of younger children also made heavier use of parent report, and therefore may also be influenced by parental mental state. Some of the included studies (e.g. Wright et al., 2021) attempted to adjust for this, but others did not. Research with the UK Understanding Society survey found those living with younger children were one of the groups experiencing the greatest deterioration in mental health during the early part of the pandemic (Pierce et al., 2020). Whilst we grouped studies by informant for meta‐analyses, the influence of different informants may play a role more widely in the heterogeneity of the findings. For example, studies using parent report are likely to be less accurate than self‐report for internalising difficulties, and vice versa, and this is likely to vary with age (Aebi et al., 2012). Interestingly, our meta‐analysis of child‐reported emotional difficulties found evidence of deterioration, whilst the corresponding meta‐analysis using parent report found no evidence of change (Figures 8 and 9). Whilst we compared data from the same informant at both time points, none of the included studies used teacher rating. Teacher ratings provide an important perspective as they have a wide range of reference to assess the level of children's difficulties, and in addition, they observe children's behaviours in a different context, which is especially important in the assessment of hyperactivity, inattention and impulsivity (Johnson, Hollis, Marlow, Simms, & Wolke, 2014; Lappalainen, Savolainen, Sointu, & Epstein, 2014).

This point relates to another consideration regarding measurement of mental health in the pandemic context. Development of all the validated mental health assessments meeting our inclusion criteria occurred prior to Covid‐19. However, the restrictions on in‐person social interaction, and changes to school, home and wider social contexts may have affected the meaning of items on some measures and made it more challenging for informants to interpret the questions as originally intended, or to calibrate their responses. The most obvious examples of this would be for measures of social behaviours, but this may also affect other domains in different ways. The mixed picture across measures suggests that any impact may be more likely to cause additional ‘noise’ in the findings rather than introduce systematic over‐ or ‐under‐estimation of change. However, currently, the extent to which existing measures retained full meaning and validity in the pandemic context is uncertain.

### Depth and strength of the literature

This discussion has highlighted the varying and sometimes inconsistent results of our included studies. As previously mentioned, different cultural and societal settings and levels of restriction are likely to be contributing factors, as on average, deterioration of mental health appeared to be more likely to be reported with higher restriction stringency levels. This association of restriction stringency with psychosocial distress has also been reported in a recent analysis of policy stringency and distress in adults, using data from 15 countries (Aknin et al., 2022). We have also alluded to other explanations which relate to individual study design and methods. Many studies included nonprobability samples recruited from education settings, where the representativeness was poorly described. Even cohort studies which were assembled to be representative are likely to have experience differential attrition during follow‐up. For example, recent analysis from the Avon Longitudinal Study of Parents and Children (ALSPAC) cohort demonstrated that a number of socio‐demographic variables were associated with being sent a questionnaire, returning a questionnaire, and completion of questionnaire during Covid‐19 follow‐ups (Fernández‐Sanlés et al., 2022). Czeisler, Wiley, Czeisler, Rajaratnam, and Howard (2021) have also argued that aspects of ‘survivorship bias’, are not fully addressed by strategies to address differential attrition, and hence many longitudinal studies provide ‘overly optimistic interpretations’ of trends. This might suggest that the relatively small effect sizes we report could be underestimates.

One of the main messages of this review for researchers is the need for improved reporting of epidemiological studies. Despite the existence of the Strengthening the Reporting of Observational Studies in Epidemiology (STROBE) guidance (von Elm et al., 2007), description of important aspects of study design, sample characteristics and findings were often poor or even missing. This may reflect the opportunistic nature of much of the research we found. Some studies included only cursory description of baseline recruitment and sample. Very few provided clear statements about representativeness and compared their sample to the general population in their setting. This meant that even fewer studies could be assessed as being representative of their population, when in some cases this may be due simply to poor reporting. Many did not provide basic descriptive statistics even in supplementary material, instead presenting only the findings of their more complex analyses.

### Research gaps and limitations

The nature of the included studies was also striking. In our searches, there was a predominance of cross‐sectional studies with single pandemic follow‐up waves, often using surveys conducted in schools or other educational settings. There was also a relative lack of studies examining externalising conditions, or which included pre‐school or early primary‐aged children, which may relate to the use of educational settings. There were few high‐quality studies which used data from representative longitudinal cohorts. Our findings here chime with the editorial by Cortese in this journal, which commended the efforts of those conducting empirical research amid the challenges of the pandemic but noted the need for continued longitudinal follow‐up of existing cohorts (Cortese, Sabe, & Solmi, 2022).

We also cannot claim that this review provides a global overview of the impact of Covid‐19. The geographic distribution of studies leaves gaps across and within whole continents. This reflects the confinement of our searches to English‐language studies and to the major databases but is also likely to be influenced by resource constraints worsened by the pandemic, where researchers may have been directed elsewhere (Cortese et al., 2022; Kroese et al., 2021).

The main limitations of our methods, other than those already mentioned, largely relate to the heterogeneity of the peer‐reviewed publications on this topic. Whilst some attempts could be made to group studies together based on design, measures, and age groups for narrative synthesis, there were further complexities in terms of widely varying lengths of follow‐up, duration of data collection throughout the pandemic, and most strikingly in the timing of data collection in respect to restrictions. Such diversity required decisions to be made in how data were presented, as many different breakdowns were possible, and limited the number of studies that could be meaningfully combined using meta‐analysis.

One of these decisions was our choice to perform meta‐analysis only for studies that used the same measure, rather than grouping those using different measures of a given ‘domain’ such as depression. Whilst this reduced the number of potential studies in each meta‐analysis, we took this decision to maintain a methodologically rigorous approach, combining like with like as far as possible and minimising heterogeneity, rather than using a more liberal approach to combining studies. If we had found similar effect sizes across measures and informants (across meta‐analyses within one domain) this would have suggested greater consistency across studies, and potentially more precise estimates of effect could have been obtained by combining studies measuring the same domain. However, as we have noted in the findings of the depression meta‐analyses, this was often not the case, and we found different results for studies using different measures within the same domain. We therefore consider that our approach has allowed us to examine nuanced differences across and within domains of mental health and produced a finer‐grained understanding of the evidence.

We have commented above on some of the evidence from individual studies which suggests differential effects by age or gender. However, due to the breadth of the literature, as well as the inconsistency of reporting, we did not conduct a formal analysis of moderators, or subgroup analyses by age, gender, ethnicity or other factors. These may act, or interact, in complex ways as risk or protective factors, and we could not do this justice in our review. For example, we did not explore the role of factors such as pre‐existing conditions or socio‐economic vulnerabilities. We note that, where reported, findings were varied. For example, Wright et al. (2021) appeared to describe a ‘levelling down’ effect early in the pandemic in their cohort study in a relatively more deprived area in England, whereby rates of maternal and child depression increased in less deprived families only but remained stable in those experiencing higher deprivation. On the other hand, two other UK studies collecting data later in the pandemic found evidence that those living in more disadvantaged households, or who were digitally excluded, were worse affected, which may suggest that the effect on these groups become more pronounced and inequalities more compounded as the pandemic progressed (Hu & Qian, 2021; Metherell et al., 2021). Other reviews of the literature have reported varying findings in terms of changes over time in those with pre‐existing conditions (Panchal et al., 2021). Our review specifically excluded clinical samples, which may account in part for differing findings, but the heterogeneity, and trajectories, of pre‐existing symptoms means that this may be a particularly difficult question to answer.

We also did not examine within‐pandemic change, which evidence suggests was again likely to be affected by a combination of risk and protective factors. For example, the ‘COVID‐19: Supporting Parents, Adolescents and Children during Epidemics’ (Co‐SPACE) study in the United Kingdom reported that groups of children followed differing within‐pandemic trajectories, with younger children, those with parent/carers with higher levels of psychological distress, or those with Special Educational Needs and/or Disabilities, experiencing increasing problems over time (Raw et al., 2021). Whilst this is an important topic, it added an unmanageable level of complexity to the review. Further research, and different study designs, are needed to address questions relating to varying trajectories, and the factors influencing whether improvements or deteriorations during the pandemic persist over the longer‐term.

Finally, related to this, we also highlight the rapidly evolving nature of this evidence base. Our update search, performed only four months later than our initial search, found almost 2000 new records for screening, and contributed 16 of our 51 included studies.

### Implications for research and policy

In our view, the process of sifting through this literature has highlighted the opportunistic and unfocussed approach that has often been taken to assessing the impacts of Covid‐19 on children and young people's mental health. This lack of coordination may have been influenced by the initial neglect of this topic by many governments, and the sudden move to online working and pressures on researchers which may have impeded communication and collaboration (Cortese et al., 2022). Similarly, many large epidemiological studies during the pandemic, including national surveys, lacked the usual involvement of children, young people and families in designing and conducting the research, due to compressed timescales.

As a research community, we can learn from this experience and work together with funders and governments to develop a more cohesive approach. There are several recommendations that we make. Firstly, strengthening research collaboration globally would have a broad range of benefits. From a global public health perspective, the International Society for Social Paediatrics and Child Health (ISSOP)'s position statement warns that widening inequity caused by Covid‐19 will reverse progress on the Millennium and Sustainable Development Goals in low‐income and middle‐income countries, impacting on children's rights to education and to health services, and on their mental health and wellbeing (Kyeremateng et al., 2022). Research can contribute to keeping a spotlight on progress and ensure that the effects on children and young people's mental health is not ignored. There are also benefits in terms of increasing our power to design analyses which could disentangle the relationships between public health measures in different settings at different times, and children and young people's mental health, and inform the choice and balance of restrictions. The Collaborative Outcomes study on Health and Functioning during Infection Times (COH‐FIT – www.coh‐fit.com) study is an example of a global approach, which attempts to formulate international estimates using common measures, and includes representative and nonrepresentative samples (Solmi et al., 2022).

Secondly, as in COH‐FIT, harmonisation of data collection and of measures will allow more accurate estimation of effects. We do not suggest a restricted range of measures, as measure selection should be thoughtful and reflect the question and the setting, but a consensus on a core suite of measures which could be recommended for research on pandemic impacts, now and in future pandemics. Similarly, our third recommendation relates to the need for governments and funders to support longer‐term data collection using representative samples, designed in partnership with public and policy stakeholders to ensure it is meaningful and relevant as well as robust (Hatch, Gazard, & Rose, 2020). This would prevent the need to rely on rapid and reactive data collection.

Fourthly, we also emphasise that our findings have a number of caveats and should be considered alongside the evidence from a range of other study designs. For example, whilst representative samples are essential, there is also a need for studies examining impacts and experiences in more marginalised or disadvantaged groups. The onus is on researchers and policymakers to meaningfully engage with communities that are underserved by research. A range of studies have suggested that certain groups might be differentially impacted by public health measures, such as Hawrilenko et al.'s finding that school closures in the United States were associated with racial and ethnic disparities in mental health outcomes (Hawrilenko, Kroshus, Tandon, & Christakis, 2021).

This leads us on to recommendations for policy and practice. It is challenging to make impactful or novel recommendations in a field that has been saturated by calls for improved support or funding for children and young people's mental health, and to make recommendations that apply across geographies and organisations. However, we can underline key messages for policy and practice. Globally, access to specialist mental health services and treatment for children and young people has been of concern even prior to the pandemic. Our findings suggest that the increase in difficulties may be of a relatively small ‘clinical’ magnitude, but this appears already to be translating to significantly increased demand at population level in terms of new presentations and presentations in crisis across various health systems, as the authors have seen in the United Kingdom (Iacobucci, 2022). Raballo, Poletti, Valmaggia, and McGorry (2021) have argued that services are now even more unlikely to be able to meet growing need without a step change in thinking by those funding and designing services. Further upstream, Hefferon et al. have described the pandemic as ‘a systemic shock to the wider determinants of child health, with impacts on family functioning and income, access to healthcare and education’ (Hefferon et al., 2021). This speaks to the need to commit to policies which are not short‐term catch‐up ‘recovery’ fixes, but which continue to support universal and targeted mental health prevention and promotion measures in family, school and community settings, including mitigation of the impact on wider social and economic structures.

Finally, at the start of the pandemic, governments had to rapidly attempt to balance risks and benefits with little evidence to inform their decisions. This is no longer the case. We now have the evidence to argue that children and young people's mental health must be explicitly considered and included in planning for any future pandemic response. Whilst high or uncontrolled levels of infections across society are detrimental to children on a variety of levels, and can place them at risk, public health protections must be carefully targeted and regularly reviewed, to minimise unintended consequences. As a research community, we must continue to study the longer‐term impacts on children and young people, and advocate for their needs.

## Supporting information


**Table S1.** Medline Search Strategy.
**Table S2.** Tables of individual study findings.

**Figure S1.** Risk of Bias assessment.
