# Supplementary material for: Annual Research Review: The impact of Covid‐19 on psychopathology in children and young people worldwide: systematic review of studies with pre‐ and within‐pandemic data
Source: J Child Psychol Psychiatry. 2022 Nov 24;64(4):611–40. doi: 10.1111/jcpp.13716 (PMC10952503; doi:10.1111/jcpp.13716)
Supplement: Supplementary file 1 — Table S1. Medline Search Strategy. Table S2. Tables of individual study findings. [file JCPP-64-611-s002.docx]

## Table S1: Medline Search Strategy

| 1 | (COVID-19 or SARS-CoV-2).af. |
| --- | --- |
| 2 | ((coronavirus or betacoronavirus or coronavirus infections) and (disease outbreaks or epidemics or pandemics)).af. |
| 3 | (nCoV* or 2019nCoV or 19nCoV or COVID19* or COVID or SARS-COV-2 or SARSCOV-2 or SARS-COV2 or SARSCOV2 or SARS coronavirus 2).ab. or (nCoV* or 2019nCoV or 19nCoV or COVID19* or COVID or SARS-COV-2 or SARSCOV-2 or SARS-COV2 or SARSCOV2 or SARS coronavirus 2).ti. |
| 4 | ((coronavirus* or corona virus* or betacoronavirus*) adj3 (pandemic* or epidemic* or outbreak* or crisis)).ab. or ((coronavirus* or corona virus* or betacoronavirus*) adj3 (pandemic* or epidemic* or outbreak* or crisis)).ti. |
| 5 | 1 OR 2 OR 3 OR 4 |
| 6 | (child* or teen* or youth* or adolescen* or juvenile* or young* or boy* or girl* or minors* or preschool* or puber* or pubescen* or pupil* or grade* or student*) |
| 7 | (Mental* or mental disorder* or mental process* or mental disease* or anxiety* or depression* or mental stress*).ab. or (Mental health* or mental disorder* or mental process* or mental disease* or anxiety* or depression* or mental stress*) |
| 8 | (mental illness* anxious or depressive or post-traumatic or posttraumatic or mood* or irritability or irritable or emotional disturbance*) |
| 9 | 5 AND 6 AND (7 OR 8) |

**Table S2: Tables of individual study findings**

## Table of results from studies measuring depression

| **Author** | **Study design** | **Country** | **Child's age (indicate baseline or follow-up)** | **Sample size (at follow-up)** | **Measure** | **Comparable results reported** | **Direction of change of mental health problems** | **Key findings from paper** | **Time range from baseline-follow-up (months)** | **Months since Jan 2020 until follow-up began** | **Baseline mean/% (most proximal to 2020)** | **Baseline SD** | **Follow-up mean/%** | **Follow-up SD** |
| --- | --- | --- | --- | --- | --- | --- | --- | --- | --- | --- | --- | --- | --- | --- |
| **Continuous measures** | | | | | | | | | | | | | | |
| Li | Cross-sectional with follow-up | China | 15.87 ± 0.74 | 831 | Beck Depression Inventory | Child-report mean and SD baseline and follow-up for males and for females, | ↓↔ | Evidence of slight decrease from before the pandemic. | 3-6 | 3 | 10.74 | 1.94 | 9.01 | 1.55 |
| Shoshani | cross sectional with f/u | Israel | 13.97 (b) | 1537 | BSI depression subscale | Child report mean and SD at baseline and follow-up | ↑ | Increase in depression symptoms | 9 | 5 | 6.14 | 4.73 | 7.59 | 5.25 |
| Lane | Comparison of cross-sectional samples | Canada | 12.66 | 1380 | CDI | Child-report mean and SD at baseline and follow-up, and by gender | ↔ | No evidence of difference | 12 | 10 | 0.43 | 0.38 | 0.40 | 0.34 |
| Hollenstein | Cohort | Canada | 12.49 (Baseline) | 178 | CDI | Child-report overall, male and female mean and SD at baseline and follow-up | ↑ | Depressive symptoms increased between baseline and follow-up, the sample-wide effect was driven by females. Wide variability across individuals | 3-9 | 3 | 1.31 | 0.28 | 1.39 | 0.33 |
| De France | Cohort | Canada | 13.9 baseline 16.2 follow-up | 136 | CDI | Correlation between time 1 and time 2 scores | ↑ | Depression scores were higher than previous trajectories would have predicted. | 24-26 estimated | 5 | NR | NR | 1.52 | 0.34 |
| Mlawer | cohort | USA | 16.02 (baseline) | 96 | CDI | child report mean and SD at baseline and follow-up | ↑ | Depressive symptoms increased. Variability across individuals with some increasing and others decreasing. Correlated with change in anxiety as well | 3-11 | 5 | 1.43 | 0.33 | 1.49 | 0.34 |
| Polack | cohort | USA | 11.77 (b) | 112 | CDI 10 item | Child report mean and SD in 2019 and 2020 | ↑ | Increase in depressive symptoms from prior to during COVID, not moderated by age or gender. | 10 | 3 | 2.85 | 3.14 | 3.96 | 3.79 |
| Walters | Comparison of cross-sectional samples | USA | 12.38 | 170 | CES-D (5 item) | Child-report mean and SD baseline and follow-up | ↔ | No evidence of change in depressive symptoms, although twice as many participants displayed an increase of 50% or more than displayed a decrease of 50% or more symptoms | 12 | 11 | 8.19 | 3.90 | 8.54 | 4.18 |
| Howard | cohort | Canada | 18.4 (b) | 411 | CES-D 10 | Child report mean and SD in 2019 and 2020 | ↑ | Scores increased across the pre-pandemic autumn term but were highest in March 2020 (0.33 SD increase in symptoms of depression after pandemic start). | 4 | 3 | 1.27 | 0.67 | 1.35 | 0.67 |
| Belanger | Cohort | Canada | 14.1 SD 1.0 (baseline) | 7354 (baseline) and 1880 (follow-up) | CES-D 10 | Child-report mean and SD at baseline and follow-up (2018, 2019, 2020) | ↔ | No change in depressive symptoms. | 23-33 | 5 | 8.61 | 5.97 | 8.88 | 5.96 |
| Wang | cohort | China | 14.04 (b) | 1,790 | CES-D 20 | child report mean and SD at baseline and follow-up | ↔ | No change in depressive symptoms | 12 | 10 | 13.69 | 10.53 | 13.44 | 10.28 |
| Liao | Cross-sectional with follow-up | China | 13.4 (0.9) | 2496 | CES-D 20 | Child-report mean and SD baseline and follow-up | ↑ | Increase in depressive symptoms | 6-7 | 7 | 15.10 | 10.50 | 15.90 | 11.10 |
| He | Cohort | China | 12.5 at baseline | 1687 | CES-D 20 | Child-report mean and SD at baseline and follow-up | ↓ | Decrease in symptoms of depression in both boys and girls | 12 | 10 | 20.97 | 10.42 | 18.78 | 11.88 |
| Hamza | Cross-sectional with f/u | Canada | estimate 16-18 ("postsecondary") | 733 | CES-D revised 20 | Child-report mean and SD at baseline and follow-up | ↑ and ↓ | For those with pre-existing mental health concerns (~30% of sample who met baseline criteria) depressive symptoms decreased. For those without pre-existing concerns however, depressive symptoms increased. | 12 | 5 | 17.62 | 13.46 | 18.44 | 13.42 |
| von Soest | cross sectional surveys | Norway | 15.51 (b) | 86,597 | Depressive Mood Inventory | Child report mean and SD in 2019 and 2021 (POMP scores) | ↑ | Depressive symptoms were 2.13 percentage points higher than expected in 2021 on a % of maximum possible score (POMP) scale (β = 2.13; 95% confidence interval (CI), 0.99 to 3.27) | 24 | 12 | 36.90 | 26.80 | 37.97 | 26.68 |
| Widnall | Cross-sectional with f/u | England | 13 to 17 (b) | 603 | HADS | No data presented other than 'no change observed' | ↔ | No change observed | 8 | 5 | NR | NR | NR | NR |
| Ertanit | cohort | Switzerland | 12.6 (b) | 319 | HSC25 depression subscale | Child report mean at baseline (intercept) and change between t1 and t2 (slope) | ↑ | Increase in depression symptoms (unadjusted); girls had significantly higher increases than boys. | 11 to 12 | 8 | 1.83 | NR | 1.95 | NR |
| Jolliff | Comparison of cross-sectional surveys | USA | 14.7 at baseline | 100 baseline 134 follow-up | PHQ-8 | Child PHQ-8 score pre-COVID to COVID (adjusted model) | ↔ | No evidence of difference, even after adjusting for covariates. | 1-6 | 3 | 6.28 | 1.42 | 7.62 | 1.36 |
| Gladstone | Cross-sectional with follow-up | USA | 14.5 baseline | 228 (595 at baseline) | PHQ-9 | Child-report mean and SD at baseline and follow-up | ↑ | Depressive symptoms increased between baseline and follow-up, although overall clinical levels of depression were low. Symptoms were higher for female adolescents. | 4-7 | 5 | 3.44 | 4.55 | 4.37 | 4.88 |
| Mehus | Cross-sectional with follow-up | USA | 18.06 baseline | 727 | PHQ-9 | Child-report mean score and 95% CIs at baseline and follow-up | ↑ | Mean symptoms of depression increased. | 3-4 | 3 | 5.70 | 5.33,6.07 | 6.83 | 6.43,7.23 |
| Andreas | Cohort | Norway | 16 years | 915 | PHQ-9 A | Child-reported mean and 95% CIs at baseline and follow-up, unadjusted | ↔ | Comparable with pre-Covid counterparts (and more so after adjustment for covariates). Those who had high ‘pandemic anxiety’ had higher depression scores | 12 | 10 | 8.17 | (7.76-8.57) | 8.58 | (8.10-9.07) |
| Adachi | cohort | Japan | 9 to 12(b) | 4118 | PHQ-A | Child report mean and SD | ↓ | Linear mixed model appears to show decrease in depression scores at follow-up time points, particularly for those who did not own a smartphone | 10 | 7 | 4.14 | 0.07 | 3.84 | 0.07 |
| Luijten | Comparison of cross-sectional samples | Netherlands | 13.4 (SD 2.8) baseline | 1318 (baseline) and 813 (follow-up) | PROMIS Depression | Child-report mean T-score (mean 50 SD 10) and SD at baseline and follow-up | ↑ | Depressive symptoms increased (mean difference = 4.9; 95% CI 4.0–5.7). Increases in depressive symptoms associated with higher parent education, and negative change in work situation for parents during the pandemic. | 22-30 | 4 | 44.70 | 10.60 | 49.40 | 8.00 |
| Bignardi | Cohort | England | 8.5-8.7 (baseline) SD 0.63-0.66 | 51 | RCADS Depression | Parent report mean change and 95% CI from baseline to follow-up | ↑ | Symptoms increased relative to 18 months earlier with a medium-to-large effect. | 7-24 | 4 | 0.71 | 0.432, 0.994 | NR | NR |
| Thorisdottir | Cross-sectional repeated with some participant overlap | Iceland | 13-18 | 17525 | SCL-90 Depressed mood subscale | Child-report ages 13-18, mean change and 95% CI | ↑ | Symptoms increased across all age groups compared to the same age groups pre-pandemic, and were significantly worse for girls than boys | 32-34 | 9 | -1.78 | -1.94, -1.63 | NR | NR |
| Halldorsdottir | Comparison of cross-sectional samples | Iceland | 16-17 years | 523 | SCL-90 Depressed mood subscale | Child-report mean and SD at baseline and follow-up by gender | ↑↔ | Although both boys and girls appeared affected, girls reported a greater negative impact during COVID-19 than boys, and their depressive symptoms were above and beyond the expected nationwide scores | 22-28 (estimate) | 10 | 9.34 | 7.60 | 10.46 | 8.86 |
| Wright | Cohort | UK | 11.97 at follow-up | 164 | SMFQ | Child-report means and SDs baseline and follow-up | ↑ | The young adolescents reported a 44% (95% CI: 23%–65%) increase in symptoms of depression. Prior to the pandemic, rates child depression were greater in families experiencing higher deprivation, but changed only in less deprived families, raising their rates to those of the high deprivation group. | 3-7 | 6 | 4.80 | 5.15 | 6.59 | 6.00 |
| Wright | Cohort | UK | 11.97 at follow-up | 202 | SMFQ | Mother rated means and SDs baseline and follow-up | ↑ | As above. Mother reports indicated an increase of 71% in depressive symptoms once maternal depression levels had been accounted for. | 3-7 | 6 | 2.00 | 3.33 | 3.74 | 4.24 |
| Magson | Cross-sectional with follow-up | Australia | 14.4 (SD 9.5) at follow-up | 244 | SMFQ | Child-report whole sample, and male and female mean and SD at baseline and at follow-up | ↑ | Number of symptoms increased in both boys and girls, but changes were more pronounced in girls (age did not moderate associations). | 6-17 | 5 | 3.81 | 4.31 | 6.12 | 6.04 |
| Westrupp | Comparison of cross-sectional samples | Australia | 9 at follow-up | 349 baseline but 2365 follow-up | SMFQ | Parent report mean and SD baseline and follow-up | ↔ | Little evidence of differences in depression in children aged 12-15 years (p was 0.06) | 48-60 | 4 | 4.28 | 4.58 | 5.04 | 12.35 |
| **Categorical measures** | | | | | | | | | | | | | | |
| Li | Cross-sectional with follow-up | China | 15.87 ± 0.74 | 831 | Beck Depression Inventory | overall prevalence (%s) | ↓ | Decrease in those meeting diagnostic criteria over time | 3-6 | 3 | 35.38 | NR | 27.8 | NR |
| Andreas | Cohort | Norway | 16 years | 915 | PHQ-9 Adolescent | Child-reported %s and 95% CI for those meeting cut-off for the clinical range unadjusted | ↔ | Comparable with pre-covid counterparts (in unadjusted and adjusted models). | 12 months | 10 | 14.29 | (12.25-16.33) | 17.07 | (5.07-19.07) |
| Myhr | Comparison of cross-sectional samples | Norway | 13-16 years at baseline | 1957 | Measure derived from Hopkins Symptom Checklist (Depressive mood inventory) | Males and females percentage with high level depressive symptoms baseline to follow-up | ↔ | No difference in percentage meeting criteria for high levels of symptoms from baseline to follow-up. | Unclear, estimate 1-4 | Unclear, estimate 4 | 12.50% |  | 14.60% |  |
| Zhang | Cohort | China | 12.6 baseline | 1241 | SMFQ | Child-report prevalence at baseline and follow-up | ↑ | Increase between baseline and follow-up. | 6 | 5 | 18.5 |  | 24.9 |  |
| Wang | Cohort | China | 14.04 (b) | 1,790 | CES-D 20 | child report proportion over cut-off on CES-D | ↔ | No change in proportion with depressive symptoms | 12 months | 10 | 30% |  | 29% |  |

## Anxiety results tables

| **Author** | **Study design** | **Country** | **Child's age (indicate baseline or follow-up)** | **Sample size (at follow-up)** | **Measure** | **Comparable results reported** | **Direction of change of mental health problems** | **Key findings from paper** | **Time range from baseline-follow-up (months)** | **Months since Jan 2020 until follow-up began** | **Baseline mean/% (most proximal to 2020)** | **Baseline SD** | **Follow-up mean/%** | **Follow-up SD** |
| --- | --- | --- | --- | --- | --- | --- | --- | --- | --- | --- | --- | --- | --- | --- |
| **Continuous measures** | | | | | | | | | | | | | | |
| Hollenstein | cohort | Canada | 12.49 (b) | 146 | Beck Anxiety Inventory | Child-report mean and SD at baseline, during lockdown and post-lockdown (average score per item not total mean) | ↓ | Decrease driven by males | 3 to 9 months | 5 months | 0.73 | 0.63 | 0.52 | 0.59 |
| Wright | cohort | uk | 11.8 (fu) | 202 | Brief Spence Anxiety | Mean scores baseline and follow-up for parent (mother) report | ↔ | No change in anxiety observed | 5-7 months | 6 | 4.4 | 3.3 | 4.4 | 3.44 |
| Magson | cross sectional with f/u | Australia | 14.4 (b) | 248 | Brief Spence Anxiety, generalised anxiety subscale | Child-report mean and SD at baseline, during lockdown and post-lockdown for males and females separately. | ↑ | Increase in scores | 6 to 17 months | 5 | 4.6 | 3.74 | 5.1 | 4.05 |
| Shoshani | cross sectional with f/u | Israel | 13.97 | 1537 | BSI anxiety subscale | child report mean and SD at baseline and follow-up | ↑ | Increase in scores | 9 months | 5 | 3.93 | 2.68 | 5.24 | 3.14 |
| Wright | cohort | UK | 11.8 (fu) | 202 | Child Trauma Scale | Child rated means and SDs baseline and follow-up | ↑ | Increase of 26% (95% CI [12%–40%]) for post-traumatic stress disorder, with corresponding maternal reports of child symptoms of 71% (95% CI [44%–99%]) and 43% (95% CI 29%–86%). | 5-7 months | 6 | 3.89 | 4.07 | 4.81 | 4.5 |
| Wright | cohort | UK | 11.8 (fu) | 202 | Child Trauma Scale | Parent rated means and SDs baseline and follow-up | ↑ | Increase of 26% (95% CI [12%–40%]) for post-traumatic stress disorder, with corresponding maternal reports of child symptoms of 71% (95% CI [44%–99%]) and 43% (95% CI 29%–86%). | 5-7 months | 6 | 1.41 | 2.28 | 2.24 | 2.8 |
| Lane | cross cohort comparison | Canada | 12.6 (b) | 1380 | GAD on SCARDRs | Child-report mean and SD at baseline and follow-up | ↔ | No evidence of change | 12 months | 10 | 0.84 | 0.52 | 0.87 | 0.52 |
| Wang | cohort | China | 14.04 (b) | 1,790 | GAD-7 | child report mean and SD at baseline and follow-up | ↔ | no evidence of change | 12 months | 10 | 3.6 | 4.32 | 3.56 | 4.22 |
| Howard | cohort | Canada | 18.4 | 411 | GAD-7 | Child report mean and SD in 2019 and 2020 | ↔ | no evidence of change | 4 months | 3 | 1.33 | 0.84 | 1.32 | 0.85 |
| Belanger | cohort | Canada | 14.1 (b) | 2099 | GAD-7 | Child-report mean and SD at baseline (2019) and follow-up | ↔ | overall models found no deterioration | 23 to 33 months | 5 | 6.06 | 5.51 | 6.23 | 5.44 |
| Hamza | Cross-sectional with f/u | Canada | 18.5 (FU) | 773 | GAD-7 | Child-report mean and SD at baseline and follow-up | ↔ | similar for pre-existing conditions; increase for those without existing problems | 12 months | 5 | 6.68 | 6.39 | 6.39 | 5.46 |
| Jolliff | Comparison of cross-sectional surveys | US | 14.7(b) | 154 | GAD-7 | Mean scores baseline and follow-up for child-report (adjusted model), and change in mean score with SD | ↔ | no evidence of change | 1-6 months | 3 | 5.52 | 1.3 | 5.01 | 1.36 |
| Widnall | Cross-sectional with f/u | England | 13 to 17 (b) | 603 | HADS | No data presented other than 'no change observed' | ↓ | decrease from pre-pandemic to first lockdown | 8 months | 5 | 7 (median) | 4 to 11 (IQR) | 6 (median) | 3-10 (IQR) |
| Ertanir | cohort | Switzerland | 12.6 (b) | 319 | HSC25 anxiety subscale | Child report mean at baseline (intercept) and change between t1 and t2 (slope) | ↔ | No evidence of change | 11 to 12 months | 8 | 1.95 |  | 1.94 |  |
| Mlawer | cohort | USA | 16.02 (baseline) | 96 | MASC | child report mean and SD at baseline and follow-up | ↑ | reports an increase on average in anxiety symptoms | 3 to 11 months | 5 | 2.14 | 0.47 | 2.2 | 0.6 |
| Luijten | cross cohort comparison | Netherlands | 13.1 (b) | 813 | PROMIS CAT V2.0-Anxiety | Child-report mean PROMIS anxiety t-score pre to during COVID-19 restrictions | ↑ | Largest differences were found for Anxiety (mean diff 7.1) | 22 to 30 months | 4 to 5 months | 43.8 | 9.7 | 50.5 | 7.6 |
| Hamza | Cross-sectional with f/u | Canada | 18.5 (F/U) | 773 | PTSD Checklist (PCL; Blevins, Weathers, Davis, Witte, & Domino, 2015) | Child-report mean and SD at baseline and follow-up | ↔ | No evidence of change | 12 months | 5 | 1.84 | 0.87 | 1.84 | 0.87 |
| Lane | cross cohort comparison | Canada | 12.6 (b) | 1380 | PTSD on SCARDS | Child-report mean and SD at baseline and follow-up | ↔ | No evidence of change | 12 months | 10 | 0.65 | 0.55 | 0.61 | 0.54 |
| Bignardi | cohort | England | 8.7/8.5 (b) | 168 | RCADS Anxiety subscale | Child-report SMD and SD baseline to follow-up | ↔ | No evidence of change | 7 to 24 months | 4 months | NR | NR | NR | NR |
| Lane | cross cohort comparison | Canada | 12.6 (b) | 1380 | SCARDS - panic | Child-report mean and SD at baseline and follow-up | ↔ | No evidence of change | 12 months | 10 | 0.3 | 0.4 | 0.29 | 0.39 |
| Gimenez-Dasi | cross sectional with f/u | Spain | 8.5 | 206 | SENA anxiety scale | child report mean and SD at baseline and follow-up | ↔ | overall no change, but decrease shown in older age group | 2 to 5 months | 3 | 2.37 | 0.66 | 2.15 | 0.08 |
| Lane | cross cohort comparison | Canada | 12.6 (b) | 1380 | sep anxiety on SCARDs | Child-report mean and SD at baseline and follow-up | ↔ | No evidence of change | 12 months | 10 | 0.52 | 0.35 | 0.51 | 0.36 |
| Lane | cross cohort comparison | Canada | 12.6 (b) | 1380 | soc anxiety on SCARDS | Child-report mean and SD at baseline and follow-up | ↔ | No evidence of change | 12 months | 10 | 0.88 | 0.54 | 0.89 | 0.57 |
| Li | Cross-sectional with f/u | China |  | 831 | Zung Self-Rating Anxiety Scale | Child report mean and SD at baseline and wave 2 (1st pandemic fu) | ↔ | No evidence of change | 6 to 7 months | 3 | 45.8 | 1.48 | 45.2 | 0.73 |
| CATEGORICAL MEASURES | | | | | | | | | | | | | |  |
| Wang | cohort | China | 14.04 (b) | 1,790 | GAD-7 | proportion over cut-off | ↔ | No evidence of change | 12 months | 10 | 31.60% |  | 32.90% |  |
| Zhang | cohort | China | 12.6 (b) | 1241 | MacArthur Health Behaviour Questionnaire generalised anxiety scale | Child report Prevalence with anxiety baseline and follow-up | ↔ | No evidence of change | 7 months | 5 months | 13.50% |  | 15.90% |  |
| Luijten | cross cohort comparison | Netherlands | 13.1 (b) | 813 | PROMIS CAT V2.0-Anxiety | child report PROMIS anxiety score % with poor functioning or severe symptoms | ↑ | Increase in proportion meeting cut-off | 22 to 30 months | 4 to 5 months | 8.60% |  | 16.70% |  |
| Li | Cross-sectional with f/u | China |  | 831 | Zung Self-Rating Anxiety Scale | prevalence with anxiety at baseline and follow-up | ↔ | Little evidence of change | 6 to 7 months | 3 | 27.68% |  | 22.98% |  |

## Internalising

| **author** | **Study design** | **Country** | **Child's age (indicate baseline or follow-up)** | **Sample size (at follow-up)** | **Measure** | **Comparable results reported** | **Direction of change: did mental health problems increase, stay the same or decrease?** | **Key findings from paper** | **Time range from baseline-follow-up (months)** | **Months since Jan 2020 until follow-up began** | **Baseline mean/% (most proximal to 2020)** | **Baseline SD** | **Follow-up mean/%** | **Follow-up SD** |
| --- | --- | --- | --- | --- | --- | --- | --- | --- | --- | --- | --- | --- | --- | --- |
| Hanno | Cohort | USA | 4 | 567 | BASC Internalising | Parent-report mean and SD at baseline and follow-up | ↑ | Fixed effects analyses estimating within-person pandemic-induced differences in outcomes.  Internalizing behaviours increased by 0.04 points (95% CI, 0.01–  0.08),  post-lockdown. | 8 to 15 | 3 | 1.68 | 0.4 | 1.71 | 0.44 |
| Frigerio | birth cohort | Italy | 3.46 (b) | 59 | CBCL anxiety/depression subscale | Parent report mean and SD at baseline and follow-up | ↑ | Analysis of trajectories indicated increase in anxious/depressed behaviour | 1 year | 5 months | 1.89 | 1.74 | 2.85 | 2.22 |
| Khoury | Cohort | Canada | 5.15 | 68 dyads | CBCL Brief Problem Monitor Internalising | Parent-report mean and SD T-score at baseline and follow-up | ↑ | Children experienced greater mental health problems during the COVID-19 pandemic compared to before | 24 to 48 | 5 | 52.46 | 4.84 | 57.56 | 6.87 |
| Chen | Cohort | China | 10.32 | 543 | Depression, Anxiety, Stress Scale 21 (DASS-21) | Child-report mean and SD at baseline and follow-up | ↑ | Increase in psychological distress at Time 2 (mean 5 1.22 [SD 5 0.30]) compared with that at Time 1 (mean 5 0.46 [SD 5 0.49]; P < 0.001) | 5 to 6 | 3 | 0.46 | 0.49 | 1.22 | 0.3 |
| Van der Laan | Cohort | Netherlands | 14.82 | 158 | RCADS Depression and Anxiety (internalising) scores | Child-report mean and SD at baseline and follow-up | ↔ | At the follow-up, the adolescents did not report more internalizing symptoms (F(1,151) ¼ 2.152, p ¼ .144, h2 p ¼ .014) after the introduction of lockdown measures when compared with baseline assessments. There was a significant main effect of gender on internalizing symptoms (F(1,153) ¼ 12.486, p ¼ .001, h2 p ¼ .127), meaning that boys had significantly less internalizing symptoms when compared with girls, both at baseline and follow-up | 1 to 34 | 4 | 40.28 | 9.72 | 41.77 | 10.29 |
| Danuinaite | Cohort | Lithuania | 13.87 | 331 | SDQ Emotional problems subscale | Child-report mean and SD at baseline and follow-up | ↑ | Small increase in rates of emotional symptoms (M_slope_ = 0.41, p < 0.001). We found significant gender effects on intercepts of emotional symptoms (β_intercept_ = − 0.25, p < 0.001), indicating higher baseline rates of these indicators in girls, compared to boys (raw figures not reported). | 16 to 19 | 9 | 2.86 | 2.29 | 3.27 | 2.47 |
| Hu | Cross sectional followed-up | UK | 13.26 | 886 | SDQ Emotional problems subscale | Child-report mean and SD at baseline and follow-up | ↑ | Increase in emotional problems The results show a decrease in the proportion of adolescents with a low level of emotional problems | 4 to 18 | 7 | 3.22 | 2.44 | 3.45 | 2.44 |
| Koenig | Matched samples within cohort | Germany | 14.93 | 324 | SDQ Emotional problems subscale | Child-report mean and SD at baseline and follow-up | ↔ | No evidence of difference between groups | 0 to 22 | 3 | 4.56 | 2.62 | 4 | 2.55 |
| Bignardi | Cohort | England | 8.7 for school group. 8.5 for lab group | 50 | SDQ Emotional problems subscale | Parent-report change in mean score and 95% CI from baseline to follow-up | ↔ | From mixed linear effects  models with no control variables, there is a non-significant  decrease of 0.25 in SDQ emotional problems (B=−0.25, 95%CI  −0.54 to 0.05) | 7 to 24 | 4 | -0.195 | -0.480, 0.089 | NR | NR |
| Feinberg | Cross sectional | USA | 9.9 (average age of oldest child in family at follow-up) | 206 | SDQ Emotional problems subscale | Parent-report mean and SD at baseline and at follow-up; % of children in clinical range at baseline and follow-up | ↑ | Effect sizes indicated large changes for Internalizing problems (d = 1.31). Parents were 4.0 times (95% CI: 2.14–7.49) more likely to report clinical levels of Internalizing problems, respectively, during the pandemic compared to pre-pandemic levels | 39 to 4 | 4 | 1.38 | 0.31 | 1.39 | 0.36 |
| Ezpeleta | Cohort | Spain | 12 | 226 | SDQ Emotional problems subscale | Parent-report mean and SD at baseline and follow-up | ↓ | Decrease in emotional problems, effect size was small (g = 0.27) | nr | 6 | 1.21 | 1.57 | 0.82 | 1.26 |
| CATEGORICAL | | | | | | | | | | | | | | |
| Ravens-Sieberer |  | Germany | 12.25 (f) | 1585 | SDQ Emotional problems subscale | Parent report: Percentage in normal, borderline and abnormal range pre and during pandemic | ↑ | Increase in proportion in ‘abnormal’ range | 36 months approx | 5 months | 10.20% |  | 13.30% |  |

## Externalising

| **Author** | **Study design** | **Country** | **Child's age (mean at baseline)** | **Sample size (at follow-up)** | **Measure** | **Comparable results reported** | **Direction of change of mental health problems** | **Key findings from paper** | **Time range from baseline-follow-up (months)** | **Months since Jan 2020 until follow-up began** | **Baseline mean/% (most proximal to 2020)** | **Baseline SD** | **Follow-up mean/%** | **Follow-up SD** |
| --- | --- | --- | --- | --- | --- | --- | --- | --- | --- | --- | --- | --- | --- | --- |
| **CONTINUOUS** | | | | | | | | | | | | | | |
| Hanno | Cohort | USA | 4 | 567 | BASC Dysregulated | Parent-report mean and SD at baseline and follow-up | ↑ | Fixed effects analyses showed children’s externalizing dysregulated (0.11 points; 95% CI, 0.06–0.16) behaviours increased after the shutdown | 08 to 15 months | 3 | 1.85 | 0.58 | 1.9 | 0.6 |
| Hanno | Cohort | USA | 4 | 567 | BASC Externalising | Parent-report mean and SD at baseline and follow-up | ↑ | Fixed effects analyses showed children’s externalizing (0.09 points; 95% confidence interval [CI] 0.05–0.13) behaviours increased after the shutdown | 08 to 15 months | 3 | 1.62 | 0.45 | 1.65 | 0.5 |
| Frigerio | birth cohort | Italy | 3.46 | 59 | CBCL attention problems subscale | Parent report mean and SD at baseline and follow-up | ↑ | Authors state trajectory analysis showed significant increase in attention problems | 1 year | 5 months | 2.08 | 1.7 | 2.73 | 1.82 |
| Khoury | Cohort | Canada | 5.15 | 68 | CBCL Brief Problem Monitor Externalising subscale | Parent-report mean and SD T-score at baseline and follow-up | ↑ | Children experienced greater externalizing (t = 6.13, p < 0.001) problems during the pandemic compared to before the pandemic | 24-48 months | 4 | 50.95 | 2.31 | 55.3 | 6.32 |
| Danuinaite | Cohort | Lithuania | 13.87 | 331 | SDQ Conduct Problems | Child-report mean and SD at baseline and follow-up | ↑ | No change in conduct problems (Mslope = − 0.02, p = 0.852) | 17 to 19 months | 2 | 2.53 | 1.53 | 2.49 | 1.57 |
| Hu | Cross-sectional with follow up | UK | 13.26 | 901 | SDQ conduct Problems | Child-report mean and SD at baseline and follow-up | ↑ | Results from person fixed-effects regression models show that adolescents with better than-median mental health before the pandemic have experienced an increase in their conduct problems | 4 to 18 months | 6 | 2.12 | 1.62 | 1.94 | 1.64 |
| Ezpeleta | Cohort | Spain | 12 | 226 | SDQ conduct problems | Parent-report mean and SD at baseline and follow-up (separately for the two subscales) | ↑ | Stepwise regression analyses controlling by previous reports of SDQ were performed. Conduct scores increased after lockdown | Unknown - reported to have taken place during year before the pandemic | 5 | 0.98 | 1.28 | 1.23 | 1.33 |
| Koenig | Matched samples within cohort | Germany | 14.93 | 324 | SDQ conduct problems | Parent-report mean and SD at baseline and follow-up (separately for the two subscales) | ↓ | Conduct problems decreased in the post-lockdown period. Again, the effects observed on conduct problems (F(5;642)=7.29, p<0.0001; LD coef: −0.17; 95% CI [−0.32; −0.02]; p=0.026) remained significant also in fully-adjusted models. | 0 (5 days) - 22 months | 2 | 2 | 1.6 | 1.76 | 1.45 |
| Feinberg | Cross-sectional with follow up | USA | Not stated | 208 | SDQ Externalising symptoms | Parent-report mean and SD at baseline and at follow-up | ↑ | Results demonstrated that differences observed in most of the outcome variables between pre-pandemic and pandemic waves were not significantly correlated with time interval. The exception was externalizing behaviours where the correlation was significant but small (r = .20, p = .05). 12.1 in clinical ranger to 16.5% in clinical range | 3 to 40 months | 3 | 1.62 | 0.36 | 1.63 | 0.42 |
| Danuinaite | Cohort | Lithuania | 13.87 | 331 | SDQ Hyperactivity/Inattention | Child-report mean and SD at baseline and follow-up | ↑ | Small increase in rates of hyperactivity/inattention (Mslope = 0.45, p < 0.001) | 17 to 19 months | 2 | 3.39 | 2.07 | 3.84 | 2.24 |
| Hu | Cross-sectional with follow up | UK | 13.26 | 901 | SDQ Hyperactivity/Inattention | Child-report mean and SD at baseline and follow-up | ↑ | Fixed-effects regression models show that adolescents with better-than-median mental health before the pandemic have experienced an increase in their hyperactivity | 4 to 18 months | 6 | 4.26 | 2.4 | 4.25 | 2.47 |
| Ezpeleta | Cohort | Spain | 12 | 226 | SDQ Hyperactivity/Inattention | Parent-report mean and SD at baseline and follow-up | ↔ | No change | Unknown - reported to have taken place during year before the pandemic | 5 | 2.49 | 2.4 | 2.5 | 2.11 |
| Koenig | Matched samples within cohort | Germany | 14.93 | 324 | SDQ Hyperactivity/Inattention; | Child-report mean and SD at baseline and follow-up | ↔ | No significant differences between groups | 0 (5 days) - 22 months | 2 | 3.51 | 2.22 | 3.53 | 1.99 |
| Walters | Cross sectional with follow-up | USA | 12.38 | 174 (171 cog impulsivity) | Weinberger Adjustment Inventory Impulse Control scale (impulsivity) | Child-report mean and SD at baseline and follow-up | ↑ | “Modest increments” in cognitive impulsivity | 12 months | 9 | 19.52 | 7.33 | 21 | 7.17 |
| Wright | Cohort | UK | 11.8 (fu) | 202 | CBCL aggression (behaviour problems) | Parent rated  means and SDs baseline and follow-up | ↑ | Mothers reported a marked increase in behaviour problems (76%) | 5-7 months | 6 | 3.59 | 4.90 | 5.23 | 5.28 |
| **CATEGORICAL** | | | | | | | | | | | | | | |
| Ravens-Sieberer | comparison of two cross-sectional/cohort samples | Germany | 12.25 (during pandemic) | 1585 | SDQ conduct | Parent report: Percentage in normal, borderline and abnormal range pre and during pandemic | ↑ | Increase in proportion in ‘abnormal’ range | 36 months approx | 5 months | 6.60% |  | 10% |  |
| Ravens-Sieberer | comparison of two cross-sectional/cohort samples | Germany | 12.25 (during pandemic) | 1585 | SDQ Hyperactivity/Inattention | Parent report: Percentage in normal, borderline and abnormal range pre and during pandemic | ↑ | Increase in proportion in ‘abnormal’ range | 36 months approx | 5 months | 7.70% |  | 14.60% |  |

## Total or combined difficulties, peer relationship problems and prosocial behaviour

| **Author** | **Study design** | **Country** | **Child's age** | **Sample size (at follow-up)** | **Measure** | **Comparable results reported** | **Direction of change of mental health problems** | **Key findings from paper** | **Time range from baseline-follow-up (months)** | **Months since Jan 2020 until follow-up began** | **Baseline mean/% (most proximal to 2020)** | **Baseline SD** | **Follow-up mean/%** | **Follow-up SD** |
| --- | --- | --- | --- | --- | --- | --- | --- | --- | --- | --- | --- | --- | --- | --- |
| Shoshani | Cross sectional with f/u | Israel | 11-17 at baseline | 1537 | Global Severity index of Brief symptom inventory | Self reported BSI, 18 psychiatric and psychological symptom items | ↑ | GSI increased between the two measurement points | 8 months | 5 | 16.47 | 11.26 | 19.18 | 12.03 |
| Hafstad |  | Norway | 12 to 16 years at follow-up | 3572 | Hopkins Symptoms Checklist-10 (HSCL-10) | Child-report overall, male and female, age and by SES mean and SD at baseline and follow-up | ↑ | Clinical levels of anxiety and depression increased slightly from 5.5% at T1 to 6.3% at T2 | 16-17 months | 6 | 0.51 | 0.61 | 0.57 | 0.64 |
| Hussong | Cohort | USA | 12 to 16 years at follow-up | 88 | Pediatric Symptom Checklist (internalising and externalising problems) | Parent reported paediatric symptom checklist | ↑ | “associated jump” in symptomology for pre and post pandemic outbreak and discontinuity in trajectories | 72 | 5 to 19 | 0.38 | 0.27 | 0.57 | 0.3 |
| Danuinaite | Cohort | Lithuania | 12-16 at baseline | 331 | SDQ peer relationship problems | Self rated SDQ mean and SD by subgroup from baseline to follow-up | ↔ | No change in peer relationship problems | 17-19 months | 9 to 10 | 2.27 | 1.84 | 2.33 | 1.77 |
| Hu | Cross sectional | UK | 10 to 16 years at baseline | 901 | SDQ peer relationship problems | YP self rated SDQ score mean and SD pre and during pandemic | ↑ | results vary with adolescents’ pre-pandemic mental health and sociodemographic backgrounds | 4-18 months | 7 | 2.02 | 1.78 | 2.3 | 1.78 |
| Ezpeleta | Cohort | Spain | 12 at baseline | 226 | SDQ peer relationship problems | Pre-post pandemic parent SDQ score | ↑ | Peer relationship problems increased after the lockdown, medium effect size | 12 (approx as just states cohort followed every year) | 6 | 0.77 | 1.29 | 1.65 | 1.56 |
| Koenig | Comparison of two matched-pair samples within the same cohort | Germany | 12-20 years at baseline | 324 | SDQ peer relationship problems | child SDQ pre-pandemic compared to matched pair child SDQ score post-pandemic | ↔ | No change | 22 | 3 to 8 months | 2.6 | 1.66 | 2.69 | 1.62 |
| Ezpeleta | Cohort | Spain | 12 at baseline | 226 | SDQ prosocial | Pre-post pandemic parent SDQ score | ↑ | ProSocial behaviour increased after the lockdown, medium effect size | 12 (approx as just states cohort followed every year) | 6 | 1.46 | 1.57 | 2.85 | 1.93 |
| Koenig | Comparison of two matched-pair samples within the same cohort | Germany | 12-20 years at baseline | 324 | SDQ prosocial | child SDQ pre-pandemic compared to matched pair child SDQ score post-pandemic | ↔ | No change | 22 | 3 to 8 months | 8.18 | 1.67 | 8.2 | 1.59 |
| Hu | Cross sectional | UK | 10 to 16 years at baseline | 901 | SDQ prosocial | YP self rated SDQ score mean and SD pre and during pandemic | ↓ | results reveal the pandemic’s diverse impacts vary with adolescents’ pre-pandemic mental health and sociodemographic backgrounds | 4-18 months | 7 | 7.86 | 1.68 | 7.73 | 1.72 |
| Danuinaite | Cohort | Lithuania | 12-16 at baseline | 331 | SDQ prosocial | Self rated SDQ mean and SD by subgroup from baseline to follow-up | ↓ | Decrease in prosocial behaviour | 17-19 months | 9 to 10 | 7.14 | 2 | 6.94 | 2.21 |
| Ezpeleta | Cohort | Spain | 12 at baseline | 226 | SDQ total difficulties | Pre-post pandemic parent SDQ score | ↑ | Psychological symptoms for total difficulties increased significantly after the lockdown, effect size was small | 12 (approx as just states cohort followed every year) | 6 | 5.45 | 4.65 | 6.2 | 4.44 |
| Koenig | Comparison of two matched-pair samples within the same cohort | Germany | 12-20 years at baseline | 324 | SDQ total difficulties | child SDQ pre-lockdown compared to matched pair child SDQ score post-lockdown | ↔ | No evidence of change | 22 | 3 to 8 months | 12.36 | 5.39 | 11.98 | 5.03 |
| Metherell | Cohort | UK | 10 to 16 years at baseline | 1387 | SDQ total difficulties | Self reported SDQ total difficulties score | ↑ | Small changes in mental health throughout the pandemic | 72+ | 15 months | 10.7 | NR | 11.1 | NR |

## Other

| **Author** | **Study design** | **Country** | **Child's age** | **Sample size (at follow-up)** | **Measure** | **Comparable results reported** | **Direction of change of mental health problems** | **Key findings from paper** | **Time range from baseline-follow-up (months)** | **Months since Jan 2020 until follow-up began** | **Baseline mean/% (most proximal to 2020)** | **Baseline SD** | **Follow-up mean/%** | **Follow-up SD** |
| --- | --- | --- | --- | --- | --- | --- | --- | --- | --- | --- | --- | --- | --- | --- |
| Hamza | Cross-sectional with f/u | Canada | 18.52 follow-up | 733 | Adapted version of the Inventory of Statements about Self-Injury | Child-report mean and SD at baseline and follow-up | ↔ | No difference across groups | 12 | 5 | 0.18 | 0.38 | 0.2 | 0.4 |
| Zhang | Cohort | China | 12.6 | 1241 | Youth Risk Behaviour Surveillance System: Self-harm | Child-report percentage saying yes | ↑ | Prevalence of non suicidal self injury increased | 6 | 5 | 31.80% |  | 42.00% |  |
| Zhang | Cohort | China | 12.6 | 1241 | Youth Risk Behaviour Surveillance System: Suicide ideation | Child-report percentage saying yes | ↑ | Prevalence of suicide ideation increased | 6 | 5 | 22.50% |  | 29.70% |  |
| Zhang | Cohort | China | 12.6 | 1241 | Youth Risk Behaviour Surveillance System: Suicide plans | Child-report percentage saying yes | ↑ | Prevalence of suicide plans increased | 6 | 5 | 8.70% |  | 14.60% |  |
| Zhang | Cohort | China | 12.6 | 1241 | Youth Risk Behaviour Surveillance System: suicide attempts | Child-report percentage saying yes | ↑ | Prevalence of suicide attempts increased | 6 | 5 | 3.00% |  | 6.40% |  |
| Koenig | Comparison of two matched-pair samples within the same cohort | Germany | 14.93 | 324 | Paykel Suicide Scale (PSS) Suicidal ideation | % of children saying yes | ↔ | Little evidence of difference between groups | 0 to 22 | 3 | 13.58% |  | 10.19% |  |
| Koenig | Comparison of two matched-pair samples within the same cohort | Germany | 14.93 | 324 | Paykel Suicide Scale (PSS) Suicide plans | % of children saying yes | ↔ | Reporting of suicide plans decreased during-pandemic compared to pre-pandemic. | 0 to 22 | 3 | 6.48% |  | 2.16% |  |
| Koenig | Comparison of two matched-pair samples within the same cohort | Germany | 14.93 | 324 | Paykel Suicide Scale (PSS) Suicide attempts | % of children saying yes | ↓ | No significant difference between groups | 0 to 22 | 3 | 0.31% |  | 0.31% |  |
| Odd | Cross-sectional using routine data | England | N/A | 85 | Suicide, as determined by child death overview panel | relative rate of suicide in pre-pandemic and during pandemic | ↔ | Overall there is little evidence that suicide deaths were higher during-pandemic compared to pre-pandemic (RR 1.09 (0.80–1.48), p = 0.584) | N/A | NR | NR | NR | NR | NR |
| Hamza | Cross-sectional with f/u | Canada | 18.52 follow-up | 733 | McLean Screening Instrument for borderline personality disorder (MSI-BPD) | Child-report mean and SD at baseline and follow-up | ↔ | No difference across groups | 12 | 5 | 2.5 | 2.54 | 2.59 | 2.51 |
| Koenig | Comparison of two matched-pair samples within the same cohort | Germany | 14.93 | 324 | Eating Disorder Examination - Questionnaire (EDE-Q) | Child-report mean and SD at baseline and follow-up | ↔ | No difference between groups | 0 to 22 | 3 | 1.18 | 0.07 | 1.11 | 0.07 |
| Valdez-Santiago | Comparison of cross-sectional surveys | Mexico | 10 to 19 | 4,812 | Last year suicide attempts | Child-report - proportion reporting last year suicide attempts | Little evidence of change | The prevalence of suicide attempts in the previous year was similar in both surveys | N/A | 8 | N/A | NA | N/A | N/A |
