# Supplementary material for: Annual Research Review: The impact of Covid‐19 on psychopathology in children and young people worldwide: systematic review of studies with pre‐ and within‐pandemic data
Source: J Child Psychol Psychiatry. 2022 Nov 24;64(4):611–40. doi: 10.1111/jcpp.13716 (PMC10952503; doi:10.1111/jcpp.13716)
Supplement: Supplementary file 2 — Figure S1. Risk of Bias assessment. [file JCPP-64-611-s001.pdf]

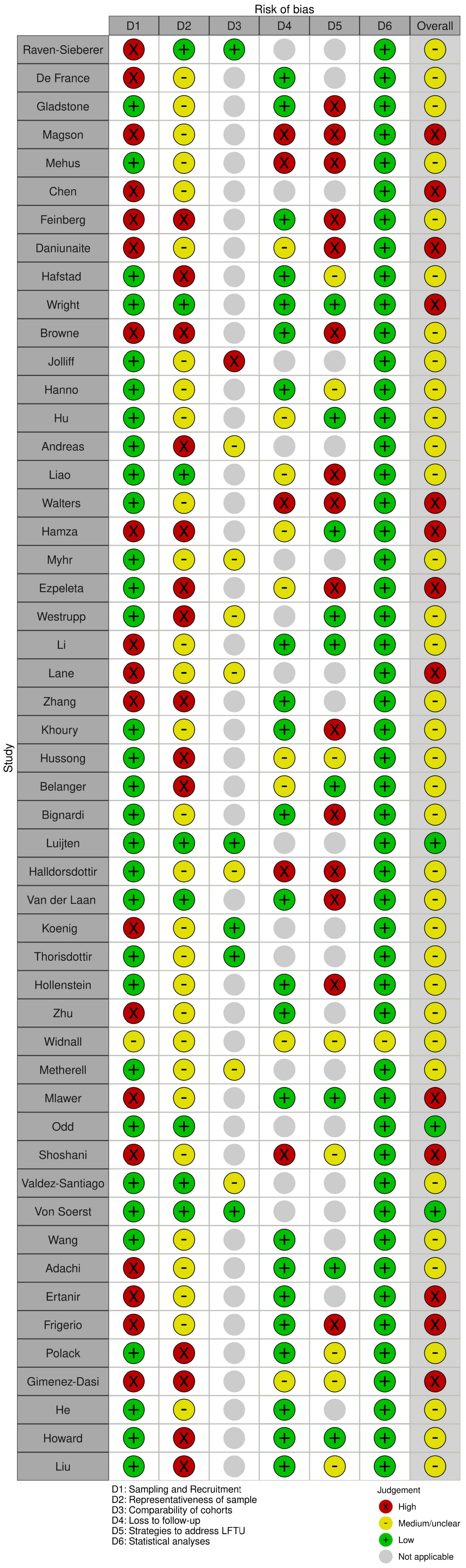
Risk of bias
D1
D2
D3
D4
D5
D6
Overall
Study
Raven-Sieberer
De France
Gladstone
Magson
Mehus
Chen
Feinberg
Daniunaite
Hafstad
Wright
Browne
Jolliff
Hanno
Hu
Andreas
Liao
Walters
Hamza
Myhr
Ezpeleta
Westrupp
Li
Lane
Zhang
Khoury
Hussong
Belanger
Bignardi
Luijten
Halldorsdottir
Van der Laan
Koenig
Thorisdottir
Hollenstein
Zhu
Widnall
Metherell
Mlawer
Odd
Shoshani
Valdez-Santiago
Von Soerst
Wang
Adachi
Ertanir
Frigerio
Polack
Gimenez-Dasi
He
Howard
Liu
D1: Sampling and Recruitment
D2: Representativeness of sample
D3: Comparability of cohorts
D4: Loss to follow-up
D5: Strategies to address LFTU
D6: Statistical analyses
Judgement
High
Medium/unclear
Low
Not applicable
